# Alternative Splicing Underpins the ALMT9 Transporter Function for Vacuolar Malic Acid Accumulation in Apple

**DOI:** 10.1002/advs.202310159

**Published:** 2024-03-21

**Authors:** Chunlong Li, Srinivasan Krishnan, Mengxia Zhang, Dagang Hu, Dong Meng, Janin Riedelsberger, Laura Dougherty, Kenong Xu, Miguel A. Piñeros, Lailiang Cheng

**Affiliations:** ^1^ Horticulture Section, School of Integrative Plant Science Cornell University Ithaca NY 14853 USA; ^2^ National Key Laboratory for Germplasm Innovation & Utilization of Horticultural Crops College of Horticulture and Forestry Sciences Huazhong Agricultural University Wuhan 430070 China; ^3^ Boyce Thompson Institute Ithaca NY 14853 USA; ^4^ Center for Bioinformatics, Simulation and Modeling, Department of Bioinformatics, Faculty of Engineering University of Talca Talca 3460000 Chile; ^5^ Horticulture Section, School of Integrative Plant Science, New York State Agricultural Experiment Station Cornell University Geneva NY 14456 USA; ^6^ Plant Biology Section, School of Integrative Plant Science and Robert W. Holley Center for Agriculture and Health USDA‐ARS Cornell University Ithaca NY 14853 USA

**Keywords:** ALMT9, alternative splicing, apple, fruit acidity, malate transport

## Abstract

Vacuolar malic acid accumulation largely determines fruit acidity, a key trait for the taste and flavor of apple and other fleshy fruits. *Aluminum‐activated malate transporter 9* (*ALMT9*/*Ma1*) underlies a major genetic locus, *Ma*, for fruit acidity in apple, but how the protein transports malate across the tonoplast is unclear. Here, it is shown that overexpression of the coding sequence of *Ma1* (*Ma1α*) drastically decreases fruit acidity in “Royal Gala” apple, leading to uncovering alternative splicing underpins Ma1's function. Alternative splicing generates two isoforms: Ma1β is 68 amino acids shorter with much lower expression than the full‐length protein Ma1α. Ma1β does not transport malate itself but interacts with the functional Ma1α to form heterodimers, creating synergy with Ma1α for malate transport in a threshold manner (When Ma1β/Ma1α ≥ 1/8). Overexpression of *Ma1α* triggers feedback inhibition on the native *Ma1* expression via transcription factor MYB73, decreasing the Ma1β level well below the threshold that leads to significant reductions in Ma1 function and malic acid accumulation in fruit. Overexpression of *Ma1α* and *Ma1β* or genomic *Ma1* increases both isoforms proportionally and enhances fruit malic acid accumulation. These findings reveal an essential role of alternative splicing in ALMT9‐mediated malate transport underlying apple fruit acidity.

## Graphical abstract

Alternative splicing of *aluminum‐activated malate transporter 9* (*ALMT9*/*Ma1*) generates two isoform proteins in apple: Ma1β being 68 amino acids shorter with much lower expression than the full‐length protein Ma1α. Ma1β does not transport malate itself, but interacts with the functional Ma1α to form heterodimers, creating synergy for vacuolar malate transport underlying apple fruit acidity.

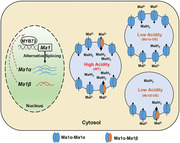

## 1 Introduction

Vacuolar accumulation of malic acid, the predominant organic acid in apple (*Malus domestica*) and many other fleshy fruits, largely determines fruit acidity, a key trait for fruit taste and flavor.^[^ ^1^ ^]^ Apple fruit acidity underwent extensive selection in its domestication process.^[^ ^2^ ^]^ During apple fruit development, malic acid level reaches the highest at the end of cell division, gradually declining to maturity.^[^ ^3^ ^]^ Malic acid accumulates in the vacuole via facilitated diffusion. Upon entry into the vacuole, malate gets protonated instantly due to the low pH generated by tonoplast H^+^‐ATPase and H^+^ pyrophosphatase.^[^ ^4^ ^]^ This “acid trapping” effectively maintains the malate concentration gradient between the cytosol and the vacuole, driving its continuous diffusion across the tonoplast.^[^ ^1^, ^5^ ^]^

Two members of the aluminum‐activated malate transporter (ALMT) family, ALMT9 and ALMT6, as well as a dicarboxylic acid transporter mediate malate transport across the tonoplast in Arabidopsis (*Arabidopsis thaliana*).^[^ ^5^, ^6^ ^]^ ALMT9 also functions as a malate‐activated chloride channel involved in controlling stomatal opening in Arabidopsis.^[^ ^7^ ^]^ Activated by cytosolic Ca^2+^, ALMT6 facilitates the bidirectional transport of malate in guard cells depending on vacuolar pH.^[^ ^5b^ ^]^ An AtALMT9 homolog in grapes (*Vitis vinifera*), VvALMT9, mediates both malic acid and tartaric acid accumulation in grape berries.^[^ ^8^ ^]^ In tomato (*Solanum lycopersicum*), *SlALMT9* is largely responsible for fruit malate levels.^[^ ^9^ ^]^ In apple, *MdALMT9* underlies *Ma*, a major QTL for fruit acidity, and was subsequently named *Ma1*.^[^ ^10^ ^]^ A natural mutation at base 1455 leads to a premature stop codon that truncates the Ma1 protein by 84 amino acids to ma1.^[^ ^2^, ^10^, ^11^ ^]^ This truncation significantly reduces Ma1's malate transport activity by disrupting a conserved C‐terminal domain, leading to low fruit acidity in recessive homozygous *ma1ma1* genotypes.^[^ ^12^ ^]^ However, the structural basis for ALMT9's malate transport function is still not fully understood. Earlier work on AtALMT9 suggests it functions as a tetramer,^[^ ^13^ ^]^ but recent cryo‐EM structural analyses indicate that both Arabidopsis ALMT1 and soybean (*Glycine max*) ALMT12, two plasma membrane‐localized ALMTs, assemble as dimers for cellular malate export.^[^ ^14^ ^]^

ALMT9 is regulated at the transcriptional level. A WRKY transcription factor, SlWRKY42, represses the expression of *SlAMT9* by binding to its promoter, and a 3‐bp deletion in the promoter disrupts this binding, releasing *SlAMT9* from transcriptional repression to confer a high acid phenotype.^[^ ^9^ ^]^ In apple, MYB73 interacts with a cold‐induced basic helix–loop–helix (bHLH) transcription factor, MdCIbHLH1, activating the expression of *Ma1* and genes encoding the vacuolar H^+^‐ATPase subunit and the vacuolar pyrophosphatase 1 for malate transport and vacuolar acidification.^[^ ^15^ ^]^ Both MdMYB73 and MdCIbHLH1 are degraded via ubiquitination by a BTB‐TAZ domain protein MdBT2 in response to nitrate, down‐regulating *Ma1* expression and malic acid accumulation.^[^ ^16^ ^]^ MdMYB123 also enhances the expression of *Ma1* and a P‐type ATPase‐encoding gene by binding to their promoters.^[^ ^17^ ^]^ In contrast, MdMYB44 represses the expression of *Ma1* as well as those encoding P‐type ATPase 10, vacuolar H^+^‐ATPase A3, and D2, lowering fruit malic acid accumulation.^[^ ^18^ ^]^ MdMYB21 also acts as a repressor for *Ma1* expression by binding to its promoter.^[^ ^19^ ^]^ During apple fruit ripening, WRKY31 interacts with ethylene response factor ERF72 in response to ethylene, transcriptionally repressing *Ma1* expression to decrease malic acid accumulation.^[^ ^20^ ^]^ However, it is not known if any posttranscriptional mechanism is involved in regulating ALMT9 function.

Alternative splicing regulates gene function in eukaryotes by producing more than one mRNA from the same gene.^[^ ^21^ ^]^ Alternative splicing increases transcriptome complexity, and different transcript isoforms may produce truncated proteins with altered function, stability, or subcellular localization.^[^ ^22^ ^]^ In plants, ≈40–63% of multi‐exon genes undergo alternative splicing.^[^ ^23^ ^]^ Alternative splicing plays essential roles in many biological processes in plants, such as development,^[^ ^24^ ^]^ circadian rhythm,^[^ ^25^ ^]^ metabolism,^[^ ^26^ ^]^ hormone signaling,^[^ ^27^ ^]^ and biotic/abiotic stress resistance.^[^ ^28^ ^]^ However, very few transporters underlying key traits in economically important crops have been characterized, with alternative splicing being essential to their function.

When the coding sequence of *Ma1* (*cMa1*) was overexpressed in the “Royal Gala” apple, malic acid accumulation in fruit was drastically decreased rather than increased. This surprising finding led us to uncover that *Ma1* undergoes alternative splicing. In this work, we describe the functional difference between the two isoforms generated by alternative splicing of *Ma1*, their interaction and dimerization in both plants and oocytes, and feedback inhibition of transgene overexpression on transcription of the native *Ma1* gene via an MYB transcription factor to show that alternative splicing is essential to Ma1's function for vacuolar malate transport in apple.

## 2 Results

### 2.1 Overexpression of *cMa1* Drastically Decreases Fruit Acidity in “Royal Gala” Apple

**Figure 1:**
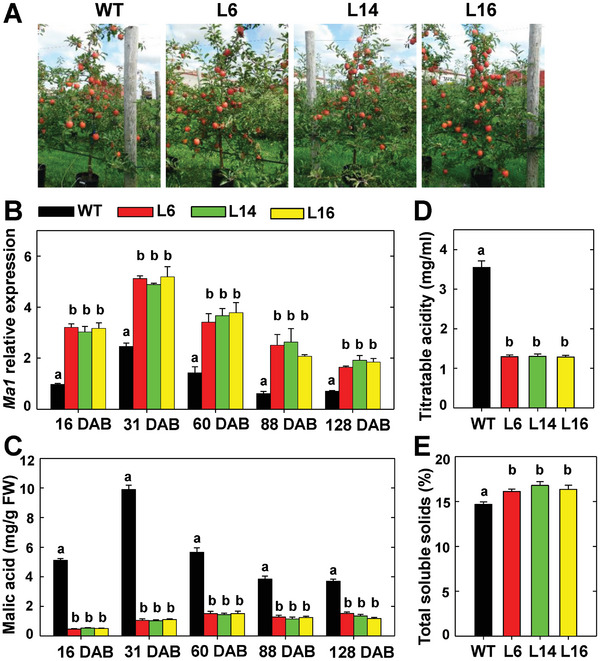
Overexpression of the *Ma1* coding sequence (*cMa1*) drastically decreases malic acid accumulation in “Royal Gala” apple fruit. A) Trees of wild type (WT) and *cMa1*‐OE transgenic lines (L6, L14, L16) at fruit harvest. B) Developmental patterns of *Ma1* expression levels in fruits of WT and *cMa1*‐OE lines measured via quantitative RT‐PCR using gene‐specific primers (Table S3, Supporting Information), with *actin* as the internal reference gene. Fruit samples were taken at 16, 31, 60, 88, and 128 days after bloom (DAB), corresponding to five key developmental stages: active cell division, end of cell division, early rapid cell expansion, late rapid cell expansion, and fruit maturity. C) Developmental changes of malic acid concentrations in fruits of WT and *cMa1*‐OE lines. D) Fruit titratable acidity of WT and *cMa1*‐OE lines at harvest. E) Fruit total soluble solids of WT and *cMa1*‐OE lines at harvest. Data in (B) to (E) are mean ± SE of five biological replicates, with six fruits pooled from two trees per replicate. Different letters (a, b) indicate significant difference between groups using Tukey's Honestly Significant Difference test at *p* < 0.05 after analysis of variance (ANOVA).

In earlier work, we showed that MdALMT9 (Ma1) localizes to the tonoplast and mediates malate transport into the vacuole when expressed in *Nicotiana benthamiana* leaves.^[^ ^10^, ^12^ ^]^ To determine the *in planta* function of Ma1, we constructed an overexpression (OE) vector of its coding sequence (*cMa1*) driven by 35S and transformed it into wild‐type (WT) “Royal Gala” apple trees. Of the over 20 independent lines generated, we selected three transgenic lines, L6, L14, and L16, with significantly higher *Ma1* expression levels in leaves, grafted them onto M.26 rootstock, and grew them for fruit acidity analysis (**Figure** **1**A). All three *cMa1*‐OE lines had significantly higher levels of *Ma1* expression compared with WT during fruit development at 16, 31, 60, 88, and 128 days after bloom (DAB), corresponding to five key developmental stages: active cell division, end of cell division, early rapid cell expansion, late rapid cell expansion, and fruit maturity (Figure 1B). We had initially predicted that these higher *Ma1* expression levels would lead to more malic acid accumulation and higher fruit acidity in the *cMa1*‐OE fruits. However, to our surprise, these transgenic fruits had much lower malic acid levels than WT throughout fruit development (Figure 1C). At fruit harvest, *cMa1*‐OE fruits had only ≈1/3 of WT titratable acidity (Figure 1D), with elevated levels of total soluble solids (Figure 1E), a response consistent with enhanced gluconeogenesis detected previously in the low acid genotype of “Usterapfel” apple.^[^ ^1^ ^]^ The drastically reduced malic acid accumulation indicates that the malate transport function of Ma1 is severely compromised in the *cMa1*‐OE fruit, which prompted us to look into the underlying molecular mechanism. As the total *Ma1* transcript level was significantly increased in the *cMa1*‐OE lines throughout fruit development, we ruled out the possibility of co‐suppression resulting from *cMa1*‐OE. This led us to postulate that posttranscriptional regulation is involved in regulating Ma1 function.

### 2.2 Ma1 Undergoes Alternative Splicing, Generating Two Isoform Proteins Ma1α and Ma1β

**Figure 2:**
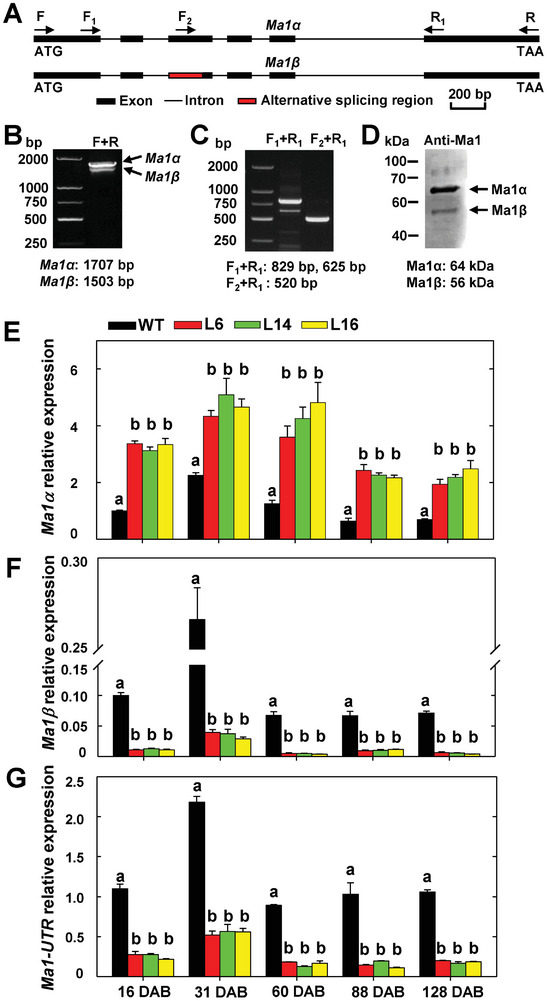
Generation of two transcript isoforms by *Ma1* alternative splicing and suppression of native *Ma1* expression by *cMa1* overexpression in “Royal Gala” apple. A) Schematic representation of the genomic structure of *Ma1* and the splicing region. The exact locations of the primers shown are marked in Figure S1 (Supporting Information). B,C) Detection of alternatively spliced *Ma1* transcripts by RT‐PCR. D) Detection of alternatively splicing‐generated Ma1 protein isoforms in WT “Royal Gala” at peak fruit acidity (31 DAB) by immunoblotting using an antibody generated against peptide ELSEKANFKDPVEA (see Figure S2, Supporting Information) in rabbit, which recognizes both Ma1α and Ma1β. E) Expression levels of *Ma1α* transcripts in the fruits of WT and *cMa1*‐OE lines (L6, L14, L16) over five developmental stages as described in Figure 1. DAB: Days After Bloom. F) Expression levels of *Ma1β* transcripts in the fruits of WT and *cMa1*‐OE lines during fruit development. G) Transcript levels of the native *Ma1* gene in the fruits of WT and *cMa1*‐OE lines during fruit development. In (E) to (G), quantitative RT‐PCR was performed using gene‐specific primers (Table S3, Supporting Information), with *actin* as the internal reference gene, and the relative expression level of each gene was obtained using the ddCT method. Data in (E) to (G) are mean ± SE of five biological replicates, with six fruits pooled from two trees per replicate. Different letters (a, b) indicate significant differences between groups using Tukey's HSD test at *p* < 0.05 after ANOVA.

The *Ma1* gene has six exons and five introns (**Figure** **2**A). By using a pair of primers targeting the coding sequence of *Ma1* on cDNAs reverse‐transcribed from RNAs of “Royal Gala” fruit taken at peak fruit acidity (31 DAB), we detected a shorter and weaker product in addition to the full‐length one via PCR (Figure 2B), suggesting the existence of alternative splicing. We designated the full‐length transcript as *Ma1α* and the shorter one as *Ma1β*, where *Ma1α* is *cMa1* or *Ma1G* named earlier.^[^ ^12^ ^]^ Based on sequencing data, we found that *Ma1β* was missing 204 nucleotides at the 5′‐end of the third exon compared with *Ma1α* (Figure 2A, Figure S1, Supporting Information). This was confirmed via PCR using two additional sets of primers, where one set led to a clearer separation of the two products while the other set yielded only one product due to the forward primer being anchored in the middle of the alternative splicing region (Figure 2C). The alternative splicing of *Ma1* led to the deletion of 68 amino acids from the full‐length protein Ma1α (Figure 2D; Figure S2, Supporting Information). Immunoblot analysis using Ma1‐specific antibody showed the shorter isoform Ma1β had a much lower expression level than the full‐length protein Ma1α in WT fruit at peak fruit acidity (31 DAB) (Figure 2D), consistent with the PCR data (Figure 2B,C).

Using qPCR primers specific to *Ma1α* or *Ma1β*, we detected their transcript levels separately during fruit development (Figure 2E,F). The transcript level of *Ma1α* was significantly higher in *cMa1‐*OE fruits than WT, but that of *Ma1β* was drastically reduced in *cMa1*‐OE fruits throughout fruit development. *cMa1*‐OE fruits had higher levels of Ma1α protein, but lower levels of Ma1β protein than WT at peak fruit acidity (Figure S3, Supporting Information). With qPCR primers targeting the untranslated regions (UTR) of the native *Ma1* gene, we found that the level of native *Ma1* transcripts was significantly lower in *cMa1*‐OE fruits compared with WT (Figure 2G), confirming that the expression of the native *Ma1* was down‐regulated in *cMa1*‐OE fruits.

### 2.3 Ma1β Localizes to Tonoplast But Does Not have Malate Transport Activity

**Figure 3:**
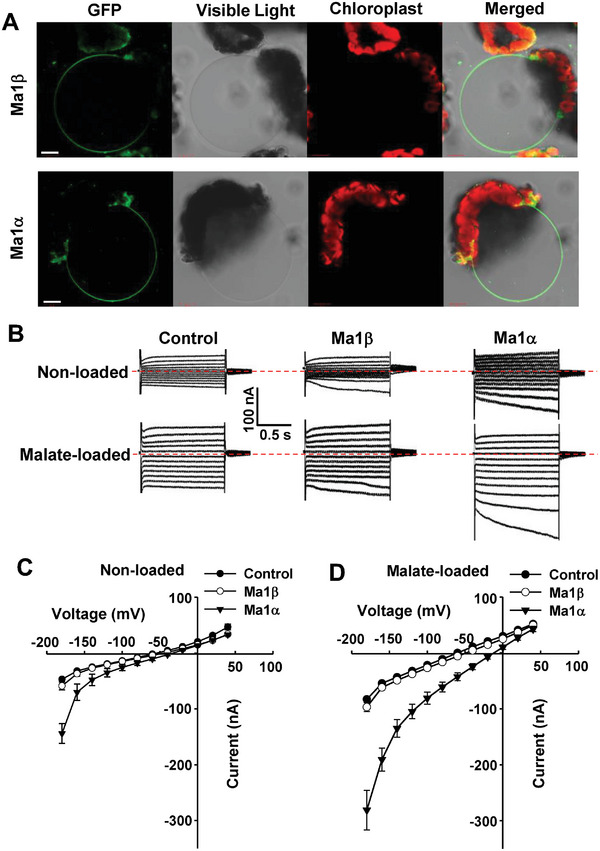
Tonoplast localization of Ma1β GFP chimera in *Nicotiana benthamiana* and functional characterization of transporter activity of Ma1β in *Xenopus laevis* oocytes. A) Ma1β‐GFP and Ma1α‐GFP in isolated vacuoles obtained after lysis of *N. benthamiana* protoplasts transiently transformed with the GFP fusion constructs. Bars = 20 µm. B) Examples of currents elicited in response to holding potentials ranging from +40 to −180 mV (in 20 mV steps) recorded in control, *Ma1β* or *Ma1α*‐expressing cells (25 ng cRNA per cell), either non‐loaded or loaded with malate by microinjecting cells with 50 nL of 100 mm Na‐Malate (increasing cytosolic malate^2−^ concentration by 4.5 mm) 2–3 h prior to the electrophysiological recordings. The red dotted line indicates the zero‐current level. C) Current–voltage (I/V) relationships constructed from steady‐state current recordings with non‐loaded cells such as those shown in (B). Data are mean ± SE. The number of cells recorded: Control (*n* = 11), Ma1β (*n* = 16), and Ma1α (*n* = 8). D) I/V relationships constructed from steady‐state current recordings with malate‐loaded cells such as those shown in (B). Data are mean ± SE. The number of cells recorded: Control (*n* = 10), Ma1β (*n* = 13), and Ma1α (*n* = 8).

Ma1α localizes to the tonoplast of plant cells and transports malate into the vacuole.^[^ ^12^ ^]^ As Ma1β has a 68‐aa deletion and its transcript level was reduced in the *cMa1*‐OE fruits, we first determined its subcellular localization relative to Ma1α. We transiently expressed *Ma1β* and *Ma1α* (as a positive control), fused with *GFP* under a 35S promoter in *N. benthamiana* leaves via agro‐infiltration. The GFP signal was localized to the tonoplast for Ma1β as its Ma1α isoform (**Figure** **3**A).

We subsequently examined Ma1β’s transport properties by expressing it heterologously in *Xenopus laevis* oocytes. The complementary RNA (cRNA) of *Ma1β* or *Ma1α*, fused with yellow fluorescent protein (*YFP*), was injected into oocyte cells, and the YFP signal was co‐localized with deep red plasma membrane (PM) marker under a confocal microscope (Figure S4A, Supporting Information). Ma1β was expressed and localized to the PM of oocytes as its Ma1α isoform. We examined the electrical properties of oocytes expressing the untagged transporters to compare the function of Ma1β and Ma1α. The resting membrane potentials (RMPs) of the cells injected with *Ma1α* cRNA were significantly less negative than those recorded in controls, whereas no difference was detected in RMPs between cells expressing *Ma1β* and controls (Figure S4B, Supporting Information). Under voltage clamp, *Ma1α*‐expressing cells mediated larger inward currents than those recorded in control cells, but the currents recorded in cells expressing *Ma1β* were not significantly different from those recorded in control cells regardless of the intracellular malate status (Figure 3B–D). These data established that Ma1β has no detectable malate transport activity, at least in oocytes under the ionic conditions tested.

### 2.4 Ma1β Interacts with Ma1α to Form a Heterodimer, Competing with Ma1α for Dimerization

**Figure 4:**
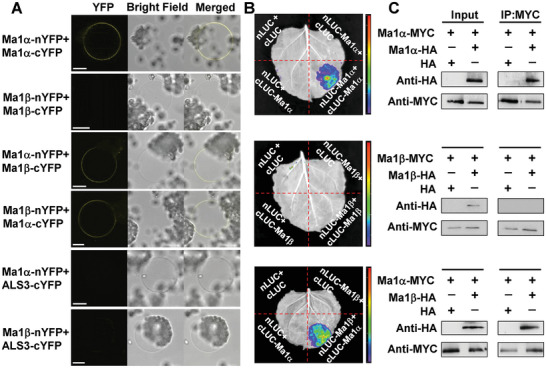
Assays of the interactions between Ma1α and Ma1β in plant cells. A) Bimolecular fluorescence complementation (BiFC) assays of interactions among and between Ma1α and Ma1β in *N. benthamiana* leaves infiltrated with *A. tumefaciens* harboring Ma1α‐nYFP/Ma1α‐cYFP, Ma1β‐nYFP/Ma1β‐cYFP, Ma1α‐nYFP/Ma1β‐cYFP or Ma1β‐nYFP/Ma1α‐cYFP fusion constructs, with a previously characterized, tonoplast‐localized protein ALS3^[^ ^29^ ^]^ as a negative control. Vacuoles were released after lysis of *N. benthamiana* protoplasts transiently transformed with the constructs indicated. Bars = 15 µm. B) Luciferase (LUC) complementation imaging assays of the interactions of Ma1α and Ma1β in *N. benthamiana* leaves infiltrated with the constructs indicated. Ma1α and Ma1β were fused in frame with nLUC and/or cLUC. Three independent biological replicates were performed. C) Co‐immunoprecipitation (IP) assays of the interactions among and between Ma1α and Ma1β in *N. benthamiana* leaves co‐transformed with the expression constructs indicated using MYC and HA antibodies.

Considering Ma1β co‐localizes with Ma1α to the tonoplast and both Arabidopsis ALMT1 and soybean ALMT12 assemble as homodimers,^[^ ^14^ ^]^ we explored the possible interactions among Ma1α or Ma1β subunits and between Ma1α and Ma1β in plant cells. We first used a bimolecular fluorescence complementation (BiFC) assay with a split YFP system in *N*. *benthamiana* leaves to examine these interactions (**Figure** **4**A). Following agroinfiltration, protoplasts were isolated, and vacuoles were subsequently released after lysis of the protoplasts. A YFP signal was detected in the tonoplast of leaf cells expressing the complementary Ma1αs, indicating that Ma1α monomers interact to form homomers in the tonoplast. By contrast, no YFP signal was detected when the complementary Ma1βs were expressed, suggesting that Ma1β monomers cannot interact among themselves. Co‐expression of complementary Ma1α and Ma1β yielded a YFP signal, indicating their interaction leading to heteromerization. By contrast, no YFP fluorescence was observed when co‐expressing Ma1α or Ma1β with a previously characterized tonoplast protein ALS3.^[^ ^29^ ^]^ We conducted a luciferase (LUC) complementation imaging assay to verify these interactions. Ma1α and Ma1β were both fused in frame with nLUC and cLUC and co‐expressed in *N. benthamiana* leaves via agro‐infiltration, with corresponding controls (Figure 4B). Strong LUC signals were detected in nLUC‐Ma1α + cLUC‐Ma1α and nLUC‐Ma1β + cLUC‐Ma1α, compared to no signal in the controls or nLUC‐Ma1β + cLUC‐Ma1β, confirming the interactions revealed by the BiFC assays. We also transiently expressed Ma1α‐MYC/HA and Ma1β‐HA/MYC fusion tag constructs in combination or individually with corresponding empty vector controls in *N. benthamiana* leaves via agro‐infiltration for co‐immunoprecipitation (Co‐IP) assay. Ma1α‐MYC was co‐immunoprecipitated with Ma1α‐HA or Ma1β‐HA whereas Ma1β‐MYC was not co‐immunoprecipitated with Ma1β‐HA (Figure 4C), further confirming the interactions between Ma1β and Ma1α as well as between Ma1α monomers and lack of interaction between Ma1β monomers in plant cells.

**Figure 5:**
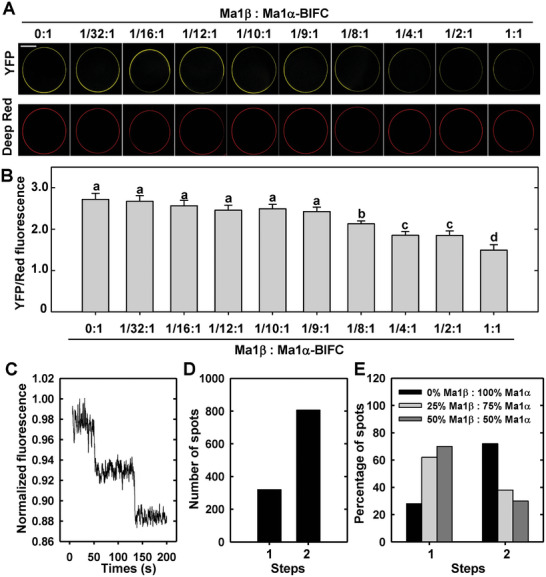
Competition of Ma1β with Ma1α for binding to Ma1α and the oligomeric state of Ma1. A) Representative images of the change in the YFP signal from Ma1α‐Ma1α bimolecular fluorescence complementation (BiFC) in oocytes (i.e., coexpression of Ma1α‐nYFP and Ma1α‐cYFP) in response to the addition of non‐tagged *Ma1β* at the indicated ratios. The total amount of *Ma1α* cRNA was fixed at 50 ng (25 ng of each complementary BiFC construct). Deep red was used as a plasma membrane marker and a reference fluorescence signal. Bar = 100 µm. B) The relative YFP/Red fluorescent signal in Ma1α‐Ma1α BiFC in response to the addition of non‐tagged *Ma1β* as shown in (A). Data are mean ± SE of 20 cells recorded at each ratio. C) Total internal reflection fluorescence (TIRF) trace showing two photo‐bleaching steps of Ma1α‐neoGreen protein complexes. The data are presented without subtracting the background fluorescence. D) Histogram showing the predominance of Ma1α‐neoGreen protein complexes displaying two photo‐bleaching steps. E) Percentages of the one or two photo‐bleaching steps observed for neoGreen‐tagged Ma1α protein complexes in oocyte cells co‐injected with 0%, 25%, or 50% of the total cRNA as untagged Ma1β.

Consistent with the observations *in planta*, Ma1α subunits interact among themselves, Ma1β subunits do not, but Ma1β interacts with Ma1α in *X. laevis* oocytes (Figure S5, Supporting Information). To examine how Ma1β competes with Ma1α subunits for oligomerization, we quantified the YFP signal resulting from the Ma1α‐nYFP + Ma1α‐cYFP BiFC interaction in response to increasing amounts of *Ma1β* cRNA co‐injected into oocytes. The YFP signal, normalized to the PM marker, decreased with increasing amounts of *Ma1β* cRNA in oocytes (**Figure** **5**A,B), indicating competition of Ma1β with Ma1α for binding to Ma1α. Significant decreases in the BiFC signal were detected at a *Ma1β* cRNA concentration of 1/8 or more of the *Ma1α* cRNA concentration co‐expressed (Figure 5B).

We proceeded to examine the in vivo stoichiometry of the oligomeric state of Ma1 using single‐molecule photobleaching step analysis to count the number of subunits making up the protein complexes. Subunit counting relies on detecting the discrete individual photobleaching steps of the fluorescently tagged Ma1 protein.^[^ ^30^ ^]^ The vast majority (≈72%) of Ma1α‐NeoGreen fluorescent spots displayed two discrete bleaching steps (Figure 5C,D), indicating that Ma1α oligomerizes as dimers. We also performed the subunit‐counting as we co‐expressed Ma1α‐neoGreen with untagged Ma1β, based on the rationale that the formation of untagged Ma1β assembly with the neoGreen‐tagged Ma1α subunits would shift the distribution from predominantly two‐step to one‐step photobleaching due to competition with Ma1α (Figure 5E). As the proportion of the total cRNA co‐injected as untagged Ma1β was increased from 0% to 25% and 50%, the Ma1α‐neoGreen dimer population decreased from ≈72% to ≈38% and ≈30%, with corresponding rises in the proportion of single step bleaching population. This shifted distribution supports the inference that Ma1α‐neoGreen and Ma1β oligomerize in vivo forming dimers.

### 2.5 Homology Modeling Reveals Structural Differences Between Ma1α‐Ma1β Heterodimer and Ma1α Homodimer

**Figure 6:**
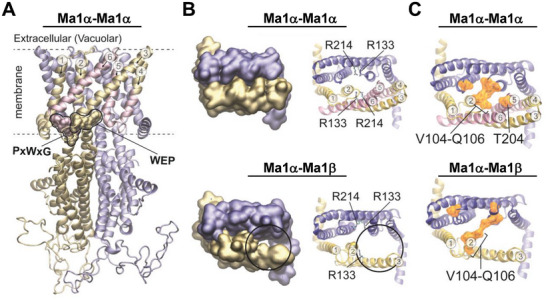
Homology modeling of the Ma1 protein structures. A) Comparative model of the Ma1α homodimer with monomers visualized in yellow and blue, respectively. Transmembrane domains missing in Ma1β are highlighted in pink. Two conserved PxWxG and WEP motifs are highlighted in the Surface representation. Yellow residues of the motifs are presented in Ma1α and Ma1β, pink residues are only presented in Ma1α. Six helices of the membrane‐spanning N‐terminal domain of the protein are numbered. Dashed lines indicate the position of the membrane. B) Top views of only the membrane‐spanning region with each monomer in a different color (blue or yellow). Represented are the Ma1α homodimer (top) and the Ma1α‐Ma1β heterodimer (bottom). On the right, arginine residues with importance for malate binding are highlighted. Four arginine residues are present in the Ma1α homodimer and three in the Ma1α‐Ma1β heterodimer. C) Top views of only the membrane‐spanning region in NewCartoon representation. Residues that are equivalent to those contributing to the extracellular gate in AtALMT1 are highlighted in orange Surface representation. In the Ma1α homodimer, V104, S105, Q106, and T204 are highlighted in each monomer. In the Ma1α‐Ma1β heterodimer, T204 is missing in Ma1β (yellow): V104, S105, and Q106 versus Ma1α (blue): V104, S105, Q106, and T204.

To gain insights into the structures of Ma1 dimers, we conducted homology modeling against Arabidopsis ALMT1 dimers.^[^ ^14a^ ^]^ In the Ma1α homodimer, the two membrane‐spanning N‐terminal protein domains, consisting of six transmembrane (TM) helices each, align parallel, forming the ion‐conducting pore, with TM2 and TM5 as pore‐ling helices (**Figure** **6**A). The missing 68 amino acids in Ma1β correspond to residues G170‐S237 that form the C‐terminal half of TM4 and the entire TM5 and TM6 (Figure 6A). In both Arabidopsis ALMT1^[^ ^14a^ ^]^ and soybean ALMT12,^[^ ^14b^ ^]^ the membrane‐spanning domain and the cytosolic domain are positioned nearly perpendicular to each other in a twisted manner. Two highly conserved motifs located at the interface, PxWxG, and WEP, are crucial for ensuring proper orientation. In Ma1α, both motifs are present and intact at positions P234‐G238 and W309‐P311 (Figure 6A). In Ma1β, however, only the WEP motif is intact, while the PxWxG motif is almost completely missing, with only the last position, G170, present. This may have affected the proper structural positioning of Ma1β, preventing the formation of Ma1β homodimers while still allowing its interaction with Ma1α.

Loss of TM5 in Ma1β makes the ion‐conducting pore bigger in the Ma1α‐Ma1β heterodimer than in the Ma1α homodimer (Figure 6B). Two arginine residues with a potential role in malate binding have been identified in Arabidopsis ALMT1,^[^ ^14a^ ^]^ which are conserved in Ma1α and correspond to R133 and R214. In the Ma1α homodimer, four arginine residues (two per monomer) loom into the center of the dimer where they may interact with malate, but only three are present in the Ma1α‐Ma1β heterodimer due to loss of R214 in Ma1β (Figure 6B).

Four residues, F51, G52, I53, and F153, constitute the extracellular gate for malate release in the AtALMT1 dimer, but they are not conserved among ALMTs.^[^ ^14a^ ^]^ Corresponding residues in Ma1α homodimer are V104, S105, Q106, T204, which occupy positions similar to the AtALMT1 dimer (Figure 6C). However, because two and a half TMs are missing in Ma1β including T204, its entire membrane‐spanning domain seems looser and more open, likely making the gate weaker in the Ma1α‐Ma1β heterodimer.

### 2.6 A Threshold Level of Ma1β is Required for Creating Synergy with Ma1α for Malate Transport

**Figure 7:**
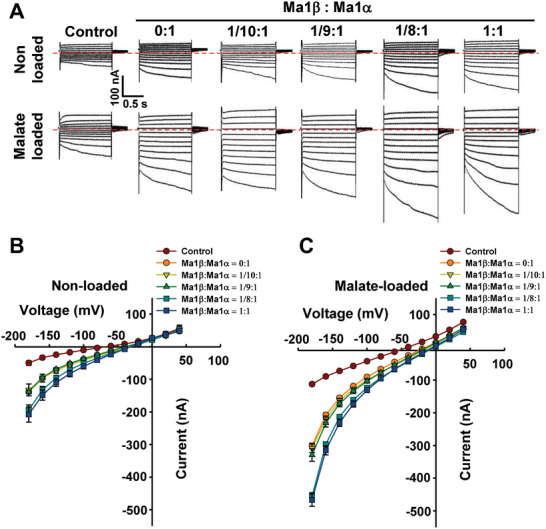
Malate transport activity in *Xenopus laevis* oocytes co‐expressing *Ma1α* and *Ma1β* genes in various ratios. A) Examples of currents elicited in response to holding potentials ranging from +40 to −180 mV (in 20 mV steps) recorded in control, *Ma1β*/*Ma1α* co‐expressing cells (Ma1: Ma1α = 0:1, 1/10:1, 1/9:1, 1/8:1, and 1:1, with the amount of *Ma1α* cRNA injected into each oocyte fixed at 25 ng), either non‐loaded or loaded with malate as in Figure 3. Zero‐current level is indicated by the red dotted line. B) I/V relationships constructed from steady‐state current recordings with non‐loaded cells such as those shown in (A). Data are mean ± SE. The number of cells recorded: Control (*n* = 10), 0:1 (*n* = 12), 1/10:1 (*n* = 12), 1/9:1 (*n* = 11), 1/8:1 (*n* = 17), and 1:1 (*n* = 10). C) I/V relationships constructed from steady‐state current recordings with malate‐loaded cells such as those shown in (A). Data are mean ± SE. The number of cells recorded: Control (*n* = 15), 0:1 (*n* = 20), 1/10:1 (*n* = 17), 1/9:1 (*n* = 12), 1/8:1 (*n* = 17), and 1:1 (*n* = 20).

As Ma1β interacts with Ma1α to form a heterodimer, we examined the effect of this interaction on Ma1α’s transport activity. We recorded the Ma1‐mediated current under voltage‐clamp in oocytes injected with a fixed amount of *Ma1α* cRNA (25 ng) and co‐injected with various amounts of *Ma1β* cRNA (**Figure** **7**; Figure S6, Supporting Information). Current amplitudes showed responses in a Ma1β‐dependent manner in both non‐loaded and malate‐loaded situations. When the amount of Ma1β was less than 1/8 of Ma1α, co‐injection of Ma1β had no effect on the currents compared with cells expressing Ma1α alone (Figure 7A–C; Figure S6A–C; Table S1, Supporting Information). Significantly larger currents were detected when the amount of Ma1β reached 1/8 of Ma1α, but any additional increase in the amount of Ma1β did not result in a further increase in currents. This ratio is similar to that for the Ma1α complex being first disrupted by Ma1β to a significant degree (Figure 5B). Across the range of holding potentials used, significant increases in the current amplitude were detected from −40 to −180 mV under malate‐loaded conditions when the ratio of Ma1β/Ma1α reached 1/8 or higher (Table S1, Supporting Information). We confirmed that the expression of Ma1α protein in the oocyte PM is not altered by co‐expression of Ma1β as illustrated by the constant signal of YFP fused to Ma1α over the range of *Ma1β* cRNA concentrations used (Figure S7, Supporting Information). These data show that Ma1β stimulates Ma1α’s transport activity, and the threshold level of Ma1β for this synergistic effect is 1/8 of Ma1α.

### 2.7 At a Constant Ratio of Ma1β to Ma1α for Synergistic Interaction, Increasing the Amount of Ma1α Co‐Expressed with Ma1β Leads to Higher Malate Transport Activity

**Figure 8:**
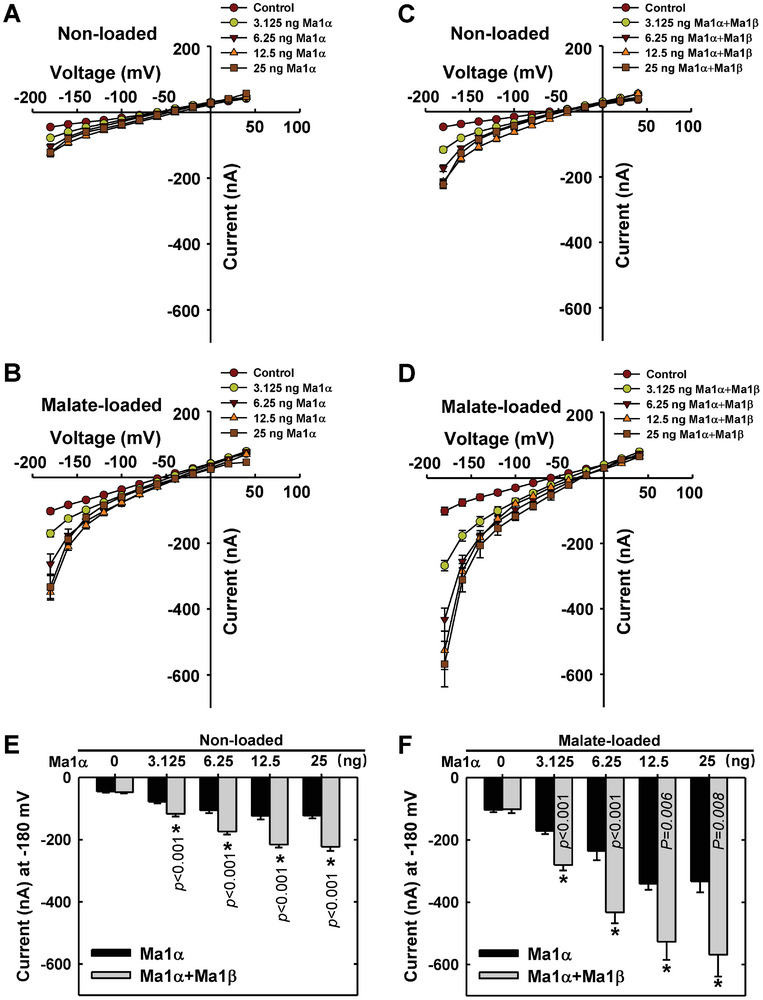
Response of malate transport activity in *Xenopus laevis* oocytes to the amount of *Ma1α* alone or co‐expressed with *Ma1β* at a constant ratio for synergistic interaction. A) I/V relationships constructed from steady‐state current recordings with non‐loaded oocyte cells (See Figure S9, Supporting Information) injected with various amounts of *Ma1α* cRNA (0–25 ng). Data are mean ± SE. The number of cells recorded for *Ma1α* cRNA at 0 (Control), 3.125, 6.25, 12.5, and 25 ng are 11, 16, 11, 10, and 10, respectively. B) I/V relationships constructed from steady‐state current recordings with malate‐loaded oocyte cells (Figure S9, Supporting Information) injected with various amounts of *Ma1α* cRNA (0–25 ng). Data are mean ± SE. The number of cells recorded for *Ma1α* cRNA at 0 (Control), 3.125, 6.25, 12.5, and 25 ng are 10, 13, 9, 10, and 9, respectively. C) I/V relationships constructed from steady‐state current recordings with non‐loaded oocyte cells (Figure S9, Supporting Information) co‐expressing *Ma1α* and *Ma1β* in a constant ratio of 1 to 1/8 at various amounts of *Ma1α* cRNA (0–25 ng). Data are mean ± SE. The number of cells recorded for *Ma1α* cRNA at 0 (Control), 3.125, 6.25, 12.5, and 25 ng are 12, 19, 20, 16, and 14, respectively. D) I/V relationships constructed from steady‐state current recordings with malate‐loaded oocyte cells (Figure S9, Supporting Information) co‐expressing *Ma1α* and *Ma1β* in a constant ratio of 1 to 1/8 at various amounts of *Ma1α* cRNA (0–25 ng). Data are mean ± SE. The number of cells recorded for *Ma1α* cRNA at 0 (Control), 3.125, 6.25, 12.5, and 25 ng are 11, 10, 11, 9, and 9, respectively. E) Response of currents to the amount of *Ma1α* cRNA in combination with *Ma1β* cRNA at −180 mV derived from (A) and (C). (F) Response of currents to the amount of *Ma1α* cRNA in combination with *Ma1β* cRNA at −180 mV derived from (B) and (D).

To determine how Ma1's malate transport activity responds to increasing amounts of Ma1α co‐expressed with Ma1β while the two have a synergistic interaction, we altered the amount of *Ma1α* cRNA co‐injected into oocyte cells with *Ma1β* cRNA while maintaining their ratio at 1 to 1/8. As this involves different amounts of Ma1β and Ma1α, we first verified that the expression of Ma1β protein in oocyte PM is not affected by co‐expression of Ma1α as shown by the constant signal of YFP fused to Ma1β over a range of *Ma1α* cRNA concentrations (Figure S8, Supporting Information). In oocyte cells injected with cRNAs of *Ma1α* alone, current amplitudes showed a curvilinear response to the amount of *Ma1α* cRNA in both non‐loaded and malate‐loaded situations (**Figure** **8**A,B; Figure S9, Supporting Information). This curvilinear response is more clearly seen for the current against the amount of *Ma1α* cRNA at −180 mV (Figure 8E,F). At low amounts, increasing the amount of *Ma1α* cRNA caused a rapid increase in the current amplitude, but it reached a plateau at 12.5 ng cRNA. In oocyte cells co‐injected with *Ma1β* cRNA, current amplitudes also exhibited a curvilinear response to the amount of *Ma1α* cRNA in both non‐loaded and malate‐loaded situations, but the values were significantly larger than those in oocyte cells injected with *Ma1α* cRNA alone at any given amount of *Ma1α* cRNA (Figure 8C–F), confirming the stimulation effect of Ma1β on Ma1α’s malate transport activity. In regions of the response curve where co‐injection of increasing amounts of *Ma1α* and *Ma1β* cRNAs led to a significant rise in the current amplitude under malate‐loaded conditions (Figure 8F), more Ma1α‐Ma1β heterodimers as well as more Ma1α homodimers were expected to form, contributing to the higher malate transport activity. At any given amount of Ma1α used, significant increases in the current amplitude were detected from −40 to −180 mV in oocytes co‐expressing Ma1β (at 1/8 of Ma1α) under malate‐loaded conditions (Table S2, Supporting Information), confirming the range of holding potentials for the stimulation of Ma1α’s malate transport activity by Ma1β observed earlier (Table S1, Supporting Information).

### 2.8 Overexpression of Either *Ma1α* or *Ma1β* Decreases Malic Acid Accumulation, Whereas Overexpression of Both *Ma1α* and *Ma1β* or Genomic *Ma1* Increases Malic Acid Accumulation in Apple

**Figure 9:**
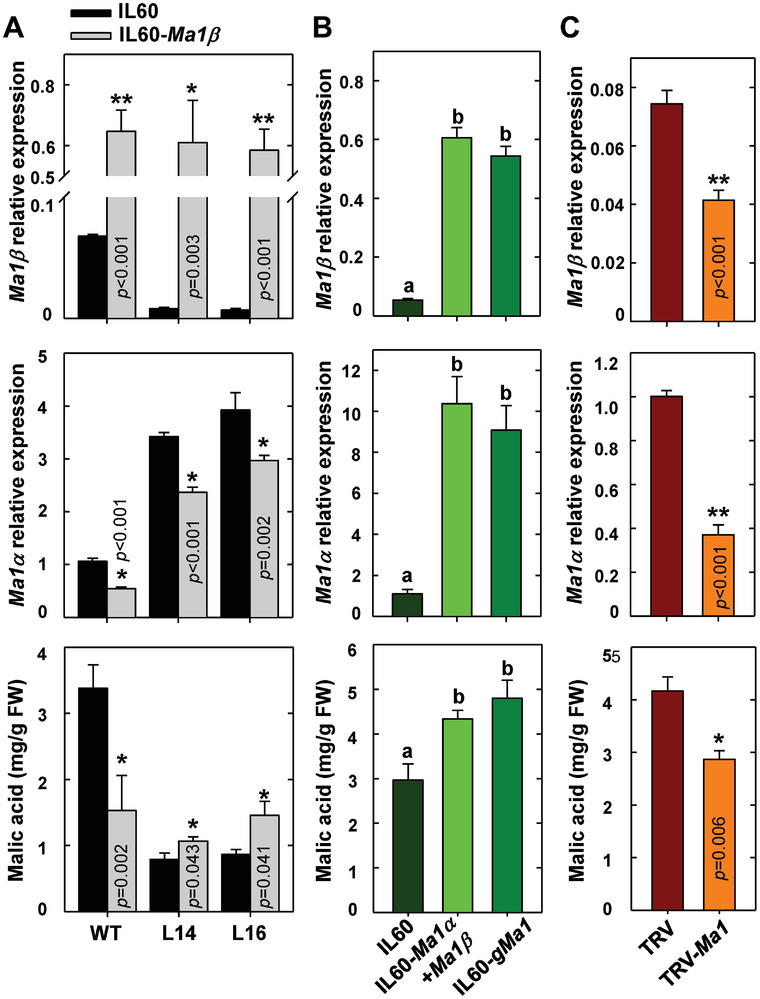
Overexpression of *Ma1β* alone, both *Ma1α* and *Ma1* or genomic *Ma1* on transcripts of *Ma1α* and *Ma1β* and malic acid levels in “Royal Gala” fruit. A) The expression levels of *Ma1β* and *Ma1α* and malic acid concentration in mature fruit after rattle virus‐based gene‐overexpression of *Ma1β* in WT and *cMa1*‐OE lines (L14 and L16). The fruit was infiltrated with virus vector IL60‐*Ma1β*, with an empty vector (IL60) as control. B) The expression levels of *Ma1β*, *Ma1,α* and malic acid concentrations after rattle virus‐based gene‐overexpression of both *Ma1α* and *Ma1β* or genomic sequence of *Ma1* (*gMa1*) in WT mature fruits. IL60‐*Ma1α* and IL60‐*Ma1β* were co‐infiltrated, or IL60‐*gMa1* was infiltrated into fruits for overexpression of *Ma1α* and *Ma1β*. Fruit infiltrated with empty vector (IL60) was used as control. C) The expression levels of *Ma1β*, *Ma1,α* and malic acid concentrations after rattle virus‐based gene‐silencing of *Ma1* in WT mature fruits. The fruit was infiltrated with *A. tumefaciens* containing TRV‐*Ma1* for silencing *Ma1* expression, with an empty vector (TRV) as control. Quantitative RT‐PCR was performed using gene‐specific primers (Table S3, Supporting Information), with *actin* as the internal reference gene, and the relative expression level of each gene was obtained using the ddCT method. Data are mean ± SE of five biological replicates with three fruits per replicate (three injection sites per fruit). * Represents significant differences using Student's *t‐test* at *p* < 0.05. Different letters (a, b, c) indicate significant differences between groups using Tukey's HSD test at *p* < 0.05 after ANOVA.

To assess the role of Ma1α and Ma1β in malic acid accumulation in apple fruit, we used virus‐based gene‐overexpression or silencing.^[^ ^31^ ^]^ We first transiently expressed *Ma1α* via IL60 vector in mature WT apple fruit, with the empty vector as control.^[^ ^31e^ ^]^ This increased the expression of *Ma1α*, but decreased the expression of *Ma1β* and reduced fruit malic acid accumulation (Figure S10, Supporting Information), which is similar to the data obtained in stably transformed *cMa1*‐OE lines (Figures 1B–D and 2F). This confirms that virus‐based transient expression can effectively verify gene function in apple fruit. Subsequently, we overexpressed *Ma1β* in WT, L14, and L16 apple fruit (**Figure** **9**A). *Ma1β*‐OE significantly increased the *Ma1β* transcript level in all three genotypes. However, analogous to the inhibition effect of *Ma1α* overexpression on *Ma1β* expression (Figure 2F), *Ma1β*‐OE lowered the *Ma1α* transcript level in WT fruit, leading to decreased malic acid accumulation. In contrast, *Ma1β*‐OE partially restored malic acid accumulation in the two *cMa1*‐OE lines (L14 and L16) by elevating the transcript level of *Ma1β* and lowering the transcript level of *Ma1α* (Figure 9A). When *Ma1α* and *Ma1β* were co‐overexpressed or the genomic *Ma1* was overexpressed in WT fruit, the transcript levels of both *Ma1α* and *Ma1β* increased proportionally, leading to higher malic acid accumulation (Figure 9B). RNAi repression of both *Ma1α* and *Ma1β* transcript levels in WT fruit significantly lowered fruit malic acid levels (Figure 9C). Taken together, these data strongly suggest that the Ma1‐mediated malic acid accumulation in apple fruit depends on the presence of both Ma1α and Ma1β, and a certain stoichiometry between the two is essential for maintaining Ma1's malate transport activity at a high level.

### 2.9 Overexpression of *cMa1* Triggers Feedback Inhibition on its Native Gene Expression via MdMYB73

**Figure 10:**
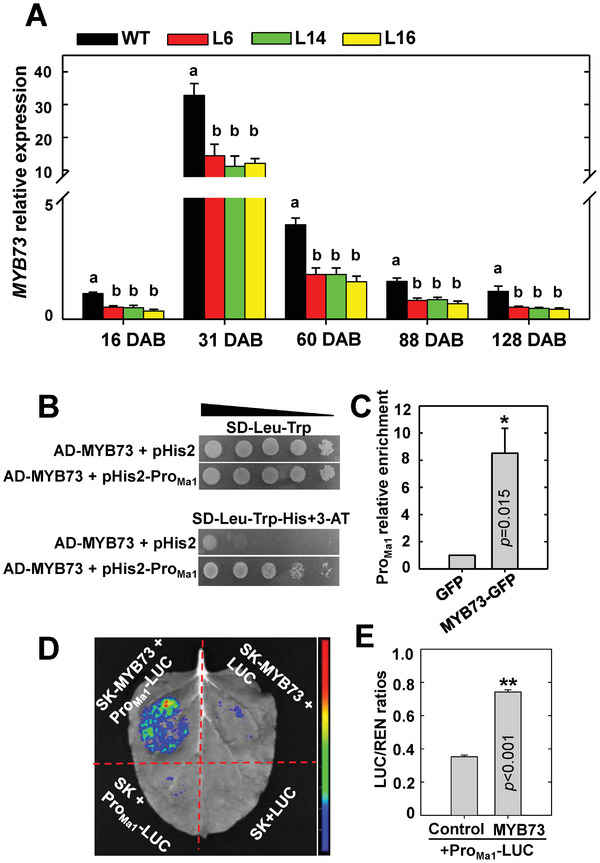
MYB73 binds to the *Ma1* promoter in regulating its expression. A) Expression levels of *MYB73* in fruits of WT and *cMa1*‐OE lines (L6, L14, L16) over five developmental stages as described in Figure 1. Quantitative RT‐PCR was performed using gene‐specific primers (Table S3, Supporting Information), with *actin* as the internal reference gene. Data are mean ± SE of five biological replicates, with six fruits pooled from two trees per replicate. Different letters (a, b) indicate significant differences between groups using Tukey's HSD test at *p* < 0.05 after ANOVA. B) Y1H assays on the binding of MYB73 protein to the *Ma1* promoter sequence, with empty vector (pHis2) as a negative control. The positive clones were cultured on SD‐Leu‐Trp‐His in the presence of 3‐AT (50 mm) over a range of yeast concentrations (10^0^ to 10^−4^) (represented by the triangle). The experiment was repeated three times. C) ChIP‐PCR confirmation of the binding of MYB73 protein to the *Ma1* promoter. The MYB73–DNA complex was co‐immunoprecipitated from MYB73‐GFP transgenic apple calli using a GFP antibody, with empty GFP vector transgenic apple calli as a negative control. Data are mean ± SE of three biological replicates, with calli grown in one petri dish as a replicate. ** Represents significant differences using Student's *t‐test* at *p* < 0.01. D) Representative image of enhanced LUC activity in *N. benthamiana* leaves as a result of MYB73 binding to the *Ma1* promoter. E) Promoter activity expressed as the LUC/REN ratio for the *Ma1* promoter‐LUC reporter in response to overexpression of *MYB73* shown in (D). Data are mean ± SE of three biological replicates, with three leaves per replicate. ** Represents significant differences using Student's *t‐test* at *p* < 0.01.

To explore the mechanism for down‐regulation of the native *Ma1* gene expression in *cMa1*‐OE lines, we first measured the transcript levels of several previously identified transcription factors that regulate the expression of *Ma1*, including *MYB21*,^[^ ^19^ ^]^ *MYB44*,^[^ ^18^ ^]^ *MYB73*,^[^ ^15^ ^]^ *MYB123*,^[^ ^17^ ^]^ and *WRKY31* and *ERF72* ^[^ ^20^ ^]^ via RT‐qPCR at peak fruit acidity (31 DAB). No difference in transcript levels was detected between *cMa1*‐OE lines and the WT for *MYB21*, *MYB44*, *MYB123*, *WRKY31*, or *ERF72* (Figure S11, Supporting Information). However, *MYB73* expression levels were significantly lower in all three *cMa1*‐OE lines (L6, L14, and L16) than in the WT throughout fruit development (**Figure** **10**A), indicating its possible involvement in the down‐regulation of the native *Ma1* expression triggered by *cMa1*‐OE. Significantly lower expression levels of *MYB73* were also detected in WT fruit transiently over‐expressing *Ma1β*, *Ma1α* + *Ma1β*, or *gMa1* relative to the empty vector controls (Figure S12A, Supporting Information) where the expression levels of the native *Ma1* gene were lowered (Figure S12B, Supporting Information). We subsequently confirmed that MdMYB73 binds directly to the *Ma1* promoter region via yeast one‐hybrid assay (Y1H) (Figure 10B) and chromatin immunoprecipitation (ChIP)‐PCR (Figure 10C). LUC assay in *N. benthamiana* showed that MdMYB73 transcriptionally activates the expression of *Ma1* (Figure 10D,E).

**Figure 11:**
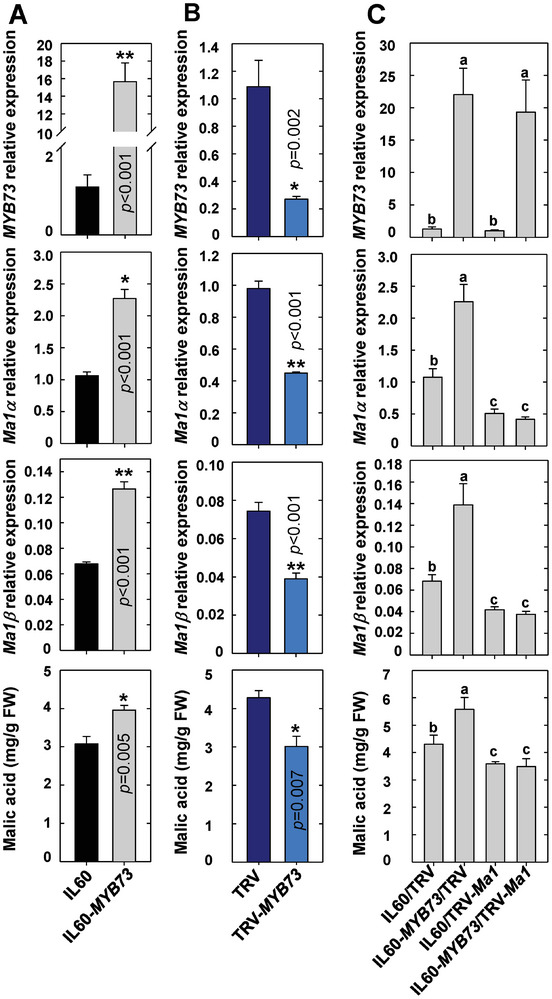
MYB73 is involved in malic acid accumulation via regulating *Ma1* expression in apple fruit. A) Expression levels of *MYB73*, *Ma1α*, *Ma1,β* and malic acid concentrations in response to rattle virus‐based overexpression of *MYB73* in mature WT fruits. Viral vector IL60‐*MYB73* was infiltrated into fruit for overexpression of *MYB73*, with empty vector (IL60) as control. B) Expression levels of *MYB73*, *Ma1α*, *Ma1,β* and malic acid concentrations in response to rattle virus‐based suppression of *MYB73* in WT mature fruits. Mature fruits were infiltrated with *A. tumefaciens* harboring TRV‐*MYB73* for silencing *MYB73* expression, with an empty vector (TRV) as control. C) Expression levels of *MYB73*, *Ma1α*, *Ma1β* and malic acid concentrations in WT mature fruits in response to overexpression of *MYB73* or suppression of *Ma1* alone or in combination via infiltration of viral vectors, IL60‐*MYB73* and *TRV‐Ma1*, alone or in combination into fruits, with empty vectors (IL60 and TRV) as control. Quantitative RT‐PCR was performed using gene‐specific primers (Table S3, Supporting Information), with *actin* as the internal reference gene, and the relative expression level of each gene was obtained using the ddCT method. Data are mean ± SE of five biological replicates with three fruits per replicate (three injection sites per fruit). * Represents significant differences using Student's *t‐test* at *p* < 0.05, ** Represents significant differences using Student's *t‐test* at *p* < 0.01. Different letters (a, b, c) indicate significant differences between groups using Tukey's HSD test at *p* < 0.05 after ANOVA.

To verify the function of MdMYB73 in regulating *Ma1* transcription in apple fruit, we transiently increased and decreased *MdMYB73* expression in WT fruit through rattle virus‐based gene‐overexpression and silencing, respectively. *MdMYB73*‐OE enhanced the transcript levels of both *Ma1α* and *Ma1β*, leading to higher malic acid accumulation (**Figure** **11**A). RNAi repression of *MdMYB73* decreased the transcript levels of both *Ma1α* and *Ma1β*, resulting in lower malic acid accumulation (Figure 11B). By using qPCR primers targeting the 5′‐UTR region of *Ma1*, we found that the transcript levels of the native *Ma1* show the same trend as the expression of *MdMYB73* in response to overexpression or RNAi of *MdMYB73* (Figure S13, Supporting Information). To confirm the regulation of malic acid accumulation by MdMYB73 goes through *Ma1*, we transiently overexpressed *MdMYB73* or repressed *Ma1* alone or in combination, with empty vectors as control. *MdMYB73*‐OE and *Ma1*‐RNAi increased and decreased the transcript levels of both *Ma1α* and *Ma1β* and malic acid concentrations in fruit, respectively, and the positive effect of *MdMYB73*‐OE on the transcript levels of both *Ma1α* and *Ma1β* and malic acid accumulation in fruit was blocked by *Ma1*‐RNAi (Figure 11C). Collectively, these data show that MdMYB73 transcriptionally activates *Ma1* expression, thereby enhancing malic acid accumulation in apple fruit. However, because the expression of *MdMYB73* was lowered in the transgenic fruit by *cMa1*‐OE, the transcript levels of both *Ma1α* and *Ma1β* from the native *Ma1* were decreased. While the decrease in the native *Ma1α* transcript was more than compensated for by *cMa1*‐OE, the ratio of *Ma1β* transcripts to the total *Ma1α* transcripts was decreased to a value well below that required for the synergistic effect of Ma1α and Ma1β, leading to much lower malic acid accumulation in the fruits of *cMa1*‐OE lines.

## 3 Discussion

In earlier work, we found that a natural mutation‐led truncation into the conserved C‐terminal domain of Ma1 by 84 amino acids to ma1 significantly reduces its malate transport function, resulting in low fruit acidity in recessive homozygous *ma1ma1* apple genotypes.^[^ ^10^, ^12^ ^]^ Here, we show that *Ma1* undergoes alternative splicing, generating a shorter isoform with deletion of 68 amino acids, Ma1β, in addition to the full‐length protein Ma1α (Figure 2). Ma1β does not have malate transport activity itself, but it interacts with the functional Ma1α to form heterodimers (Figures 3, 4, 5, 6), creating synergy with Ma1α for malate transport when Ma1β is equal to or exceeds 1/8 of Ma1α (Figure 7). Overexpression of *cMa1* (*Ma1α*) triggers down‐regulation of the native *Ma1* expression via MYB73, decreasing the Ma1β level well below the threshold that leads to significant reductions in Ma1 function and malic acid accumulation (Figures 1, 2, and 11). These findings reveal that alternative splicing underpins the function of an ALMT for determining fruit acidity, a key trait for the taste and flavor of fleshy fruits.

Ma1β does not transport malate itself in oocytes (Figure 3B–D) and is expressed at a much lower level than Ma1α in apple fruit (Figure 2E,F), but a threshold level of Ma1β, relative to Ma1α, is required to generate synergy between Ma1α and Ma1β for malate transport in oocytes (Figure 7). Ma1α and Ma1β interact to form two types of dimers, Ma1α‐Ma1β heterodimers and Ma1α homodimers. These interactions are illustrated by BiFC assays in both *N. benthamiana* and oocytes, LUC complementation imaging assays, and Co‐IP assays in *N. benthamiana* (Figure 4; Figure S5, Supporting Information). Total internal reflection fluorescence (TIRF) analyses show that 1) Ma1α primarily exists as homodimers in the absence of Ma1β (Figure 5D); and 2) the addition of Ma1β reduces the proportion of Ma1α homodimers due to its competition with Ma1α for dimerization (Figure 5E), indicating the formation of Ma1α‐Ma1β heterodimers. The formation of Ma1α homodimers is consistent with the recent findings that both Arabidopsis ALMT1 and soybean ALMT12 function as homodimers based on cryo‐EM structural analyses.^[^ ^14^ ^]^ Despite the deletion of 68 amino acid residues that form the C‐terminal half of TM4 and the entire TM5 and TM6 in Ma1α by alternative splicing (Figure 6), Ma1β still interacts with Ma1α to form heterodimers. When the amount of *Ma1β* cRNA injected into oocytes is equal to or exceeds 1/8 of *Ma1α* cRNA, Ma1α’s malate transport activity is enhanced by up to ≈60% at −180 mV (Figures 7 and 8). This suggests that Ma1α‐Ma1β heterodimers have significantly higher malate transport activity than Ma1α homodimers in oocytes. It remains to be determined why addition of more Ma1β beyond the 1/8 of Ma1α did not lead to any further increase in malate transport activity (Figure 7).

In apple fruit, the stimulation effect of Ma1β on Ma1α‐mediated malic acid accumulation estimated from the degree of increases in fruit malic acid levels during fruit development and titratable acidity at harvest in the fruit of WT relative to *cMa1*‐OE lines ranges from ≈200% at harvest to ≈500% at peak fruit acidity (Figure 1C,D). This large stimulation suggests the bulk of Ma1‐mediated malate transport in apples is fulfilled by Ma1α‐Ma1β heterodimers while Ma1α homodimers may only provide a baseline level of malate transport activity. As the levels of Ma1α and Ma1β proteins are proportional to their transcript levels in apple fruit at peak fruit acidity (Figure 2D; Figure S3, Supporting Information), the relatively constant ratio of *Ma1β* to *Ma1α* throughout fruit development in WT (Figure 2E,F) suggests that, in WT fruit, Ma1 operates at a Ma1β to Ma1α ratio close to the threshold value of 1/8 to 1 obtained in oocytes. Both stable *cMa1*‐OE and transient *Ma1α*‐OE decreased the ratio of *Ma1β* to *Ma1α* to values well below the threshold, leading to lower malate transport activity as indicated by reduced fruit malic acid accumulation (Figures 1C,D and 2E,F; Figure S10, Supporting Information). Transient *Ma1β*‐OE in the fruits of *cMa1*‐OE lines brought the ratio of *Ma1β* to *Ma1α* above the threshold, leading to partial restoration of fruit malic acid accumulation (Figure 9A). These findings corroborate the importance of having the ratio of Ma1β to Ma1α at or above the threshold value for their synergistic interaction on malate transport. However, when the ratio of Ma1β to Ma1α is at or above the threshold, the amount of Ma1α also makes a difference to the overall Ma1 function as reductions in *Ma1α* transcript levels caused by transient *Ma1β*‐OE in WT fruit led to lower malic acid accumulation (Figure 9A). This is consistent with the finding in oocytes that reducing the amount of *Ma1α* and *Ma1β* cRNAs co‐injected while keeping the ratio constant for synergistic interaction decreased Ma1's malate transport activity (Figure 8). In this case, as the amount of Ma1β is not limiting, reducing the amount of Ma1α lowers the number of Ma1α‐Ma1β heterodimers as well as Ma1α homodimers.

The membrane potential of plant vacuoles ranges from −20 to −60 mV depending on plant species and experimental conditions, with −30 mV being the most typical.^[^ ^4^, ^32^ ^]^ Although a significant stimulation of Ma1α’s transport activity by Ma1β at Ma1β/Ma1α ≥1/8 was not detected in oocytes until the holding potential reached −40 mV (Figure 7; Table S1, Supporting Information), this stimulation is physiologically relevant given the highly acidic vacuole in apple fruit cells of the *Ma1ma1* or *Ma1Ma1* genotype. Average pH values as low as 3.27 and 3.16 were detected, respectively, in bulk apple fruit of *Ma1ma1* and *Ma1Ma1* genotypes in a segregating population at fruit maturity.^[^ ^33^ ^]^ The actual pH values of the vacuole in these fruits are expected to be even lower during fruit development. These pH values are ≈4 units lower than that of the cytosol (pH 7.1 to 7.5),^[^ ^34^ ^]^ creating an equilibrium potential (Nernst potential) twice as large as that for typical plant vacuoles with a pH 5.1 to 5.5.^[^ ^4a^ ^]^ This steeper electrochemical gradient would allow apple fruit vacuoles to operate at a more negative resting membrane potential than those found in typical plant vacuoles for the accumulation of malic acid, sugars, and other metabolites. Furthermore, it is worth noting that the stimulation effect of Ma1β on Ma1α’s transport activity was characterized in oocytes, a heterologous system, and therefore, direct extrapolation of the magnitude of stimulation to the apple vacuole should be treated cautiously as unknown differences inevitably exist between the two systems. The much smaller stimulation of Ma1α’s malate transport activity by Ma1β detected in oocytes (≈60%) than that inferred from WT apple relative to *cMa1*‐OE lines (200–500%) is most likely related to differences in the constitution and configuration of the membrane and the ionic composition and gradient surrounding the transporter, with the native environment and possible presence of interacting partners in the tonoplast being more conducive to the function of the Ma1α‐Ma1β heterodimers. So when all the evidence (both in oocytes and *in planta*) is considered, the logical conclusion is that Ma1β stimulates Ma1α’s malate transport activity by forming Ma1α‐Ma1β heterodimers.

How could Ma1α‐Ma1β heterodimers have higher malate transport activity than Ma1α homodimers? Of the transmembrane transporters that form oligomers, the closest to our case is plasma membrane intrinsic proteins (PIPs) for water transport across cellular membranes. In maize (*Zea mays*), strawberry (*Fragaria x ananassa*), and other plant species, PIPs are clustered into two groups, PIP1s and PIP2s, based on their sequence similarity. Many PIP1s cannot transport water on their own due to their inability to reach the plasma membrane, whereas PIP2s are functional aquaporins by forming homotetramers. Co‐expression of maize PIP1;2 with PIP2;5 in oocytes leads to a significant increase in water transport activity.^[^ ^35^ ^]^ A similar effect is also detected between strawberry PIP1;1 and PIP2;1.^[^ ^36^ ^]^ The enhanced water transport activity results from 1) increased targeting of PIP1s to the plasma membrane and their functional activation when co‐expressed with their respective PIP2 partners and 2) enhancement of PIP2s function due to the formation of heretrotetramers with the corresponding PIP1s.^[^ ^35^, ^36^, ^37^ ^]^ ALMTs and PIPs are structurally similar in that they all have six transmembrane helices in the N‐terminal domain of their monomers, but important distinctions exist in how they function as shown by the apple ALMT9 (Ma1). First, in contrast to PIP1s, Ma1β can readily target the tonoplast (Figure 3A), but it does not oligomerize among its monomers (Figure 4; Figure S5, Supporting Information) and does not have any malate transport activity (Figure 3B–D) due to the loss of 68 amino acid residues. Second, whereas the functional unit for PIPs is a monomer,^[^ ^38^ ^]^ the malate transport channel of ALMT is formed between two monomers based on the cryo‐EM structures of both AtALMT1 and GmALMT12.^[^ ^14^ ^]^ The Ma1 dimers operate in a similar fashion (Figures 4, 5, 6). Compared with the Ma1α homodimer, however, lack of TM5 in Ma1β appears to make the ion‐conducting pore bigger in the Ma1α‐Ma1β heterodimer for malate to go through, with malate still being recognized despite only three arginine residues being present in the central cavity for malate binding in the Ma1α‐Ma1β heterodimer due to loss of R214 in Ma1β (Figure 6B,C). In addition, the loss of two and a half TMs in Ma1β including one of the two T204 residues that form the restriction of the extracellular gate (Figure 6C) is expected to make it easier for malate to be released into the vacuole. Finally, while PIP heterotetramers assemble from two types of monomers encoded by different members of the PIP gene family, the two monomers in the Ma1 heterodimer are encoded by the same gene via alternative splicing. This illustrates the functional innovation of alternative splicing in generating a truncated protein that assembles with its full‐length partner into a quaternary structure to make transmembrane transport more efficient than would otherwise be possible. The essential role *Ma1β* plays in determining Ma1's malate transport function despite being expressed at a much lower level than *Ma1α* also demonstrates that the importance of any splicing isoform cannot be solely judged on its transcript level in the transcriptome because a low‐abundance splicing transcript is not necessarily splicing noise.^[^ ^39^ ^]^

MdMYB73 is part of a feedback loop that regulates the expression of the native *Ma1* gene in response to overexpression of *Ma1* splicing forms. MYB73 binds to the promoter of *Ma1*, as demonstrated by Y1H and ChIP‐PCR assays (Figure 10B,C). The LUC assay clearly shows the transcriptional activation of *Ma1* by MYB73 (Figure 10D,E). The role of MYB73 in regulating *Ma1* expression and function in apple fruit is supported by corresponding increases and decreases in both *Ma1α* and *Ma1β* transcripts and fruit malic acid levels in overexpression and RNAi of *MYB73*, respectively, and the effect of MYB73 on malic acid accumulation being blocked by RNAi of *Ma1* (Figure **11**). In WT fruit transiently expressing *Ma1β*‐OE, *Ma1α*‐OE + *Ma1β*‐OE or *gMa1*‐OE as well as in *cMa1*‐OE lines, lower transcript levels of *MYB73* (Figure 10A; Figure S12A, Supporting Information) are most likely responsible for the down‐regulation of the native *Ma1* gene expression (Figure 2F,G; Figure S12B, Supporting Information). As this feedback inhibition on the native *Ma1α* and *Ma1β* transcripts are overcompensated for when both *Ma1α* and *Ma1β*, or *gMa1* is overexpressed, both *Ma1α* and *Ma1β* transcript levels are increased proportionally, resulting in higher Ma1 malate transport activity and higher malic acid accumulation (Figure 9B). However, when *Ma1α* or *Ma1β* is overexpressed alone, only the feedback inhibition on its corresponding native transcript is over‐compensated for, while the expression of the other isoform is decreased. This either reduces the Ma1β level well below the threshold required for synergistic interaction between Ma1α and Ma1β in the case of *cMa1*‐OE fruits (Figure 2E,F) or decreases the Ma1α level so low that it limits the formation of Ma1α‐Ma1β heterodimers as well as Ma1α homodimers in the case of *Ma1β*‐OE fruit (Figure 9A), leading to lower Ma1 malate transport activity for fruit malic acid accumulation in both cases (Figures 1C,D and 10A). How overexpression of *Ma1* splicing forms triggers down‐regulation of *MYB73* expression remains unclear and warrants further research.

**Figure 12:**
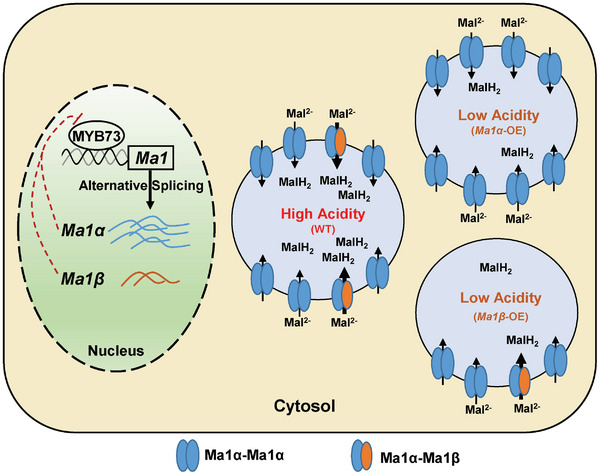
Proposed model for alternative splicing‐mediated Ma1 (ALMT9) function in transporting malate across the tonoplast in apple. Alternative splicing of *Ma1* generates two transcripts encoding two isoform proteins, Ma1α and Ma1β. Ma1β is 68 amino acids shorter with much lower expression than the full‐length protein Ma1α. They interact to form Ma1α‐Ma1β heterodimers as well as Ma1α homodimers, with the heterodimers having significantly higher malate transport activity than the homodimers. Ma1β does not transport malate itself, but a threshold level of Ma1β (Ma1β ≥1/8 of Ma1α) is required for enough Ma1α‐Ma1β heterodimers to form, generating synergy for malate transport. Ma1 operates at the threshold in wild‐type “Royal Gala” apples, maximizing its malate transport activity for vacuolar malic acid accumulation (shown at the center). Overexpression (OE) of *Ma1α* or *Ma1β* triggers feedback inhibition on the native *Ma1* expression via transcription factor MYB73, decreasing either the Ma1β level well below the threshold (Upper right) or the Ma1α level to a low value that limits the formation of the heterodimers and the homodimers (Lower right), both of which diminish Ma1's malate transport activity for malic acid accumulation.

Based on the findings presented here, we propose a model to describe the reliance of the Ma1 function on alternative splicing‐generated two isoforms, Ma1α and Ma1β (**Figure** **12**). They interact to form Ma1α‐Ma1β heterodimers as well as Ma1α homodimers, with the heterodimers having a significantly higher activity than the homodimers for malate transport across the tonoplast. Ma1β creates synergy with Ma1α for malate transport in a threshold manner when Ma1β is equal to or exceeds 1/8 of Ma1α. In the WT “Royal Gala” apple, Ma1 operates at the threshold, maximizing its malate transport activity for vacuolar malic acid accumulation with minimal investment in Ma1β. When *cMa1* (*Ma1α*) is overexpressed alone, the expression of the native *Ma1* gene is down‐regulated via MYB73, decreasing the Ma1β level well below the threshold that leads to drastic reductions in Ma1's malate transport activity and malic acid accumulation in the vacuole. When *Ma1β* is overexpressed alone, down‐regulation of the native *Ma1* gene via MYB73 decreases the Ma1α level, reducing Ma1's malate transport activity by lowering the number of Ma1α‐Ma1β heterodimers as well as Ma1α homodimers. Only overexpression of both *Ma1α* and *Ma1β* or genomic *Ma1* increases Ma1α and Ma1β proportionally, enhancing Ma1's malate transport activity for fruit malic acid accumulation. As ALMT9‐mediated malate transport underlies the fruit acidity of many fleshy fruits, it would be highly interesting to test if alternative splicing is a widespread mechanism for governing ALMT9 function. Elucidating the role of alternative splicing in ALMT9 function not only allows us to understand the molecular basis of ALMT9‐mediated malate transport but also paves the way for effectively improving the taste and flavor of apple and possibly other fleshy fruits via transgenic/gene editing approaches.

## 4 Experimental Section

### Plants and Growth Conditions

Untransformed wild‐type “Royal Gala” apple (*Malus domestica*) and three transgenic lines overexpressing the coding sequence (CDS) of *Ma1* (*cMa1*), L6, L14, and L16, were used in this study. “Royal Gala” is of heterozygous genotype (*Ma1ma1*) with respect to the “*Ma*” locus. The trees were 4 years old and grown outside on M.26 rootstock in 20‐L containers at the Cornell Experimental Orchards. They were arranged in a completely randomized design in five replicates per genotype, with two trees per replicate. The trees were maintained under standard horticultural management and disease and insect control.

Calli derived from apple cv “Orin” were used for chromatin immunoprecipitation (ChIP)‐PCR assays. The calli were cultured on Murashige and Skoog (MS) medium with 30 g L^−1^ of sucrose, 1.5 mg L^−1^ of 6‐benzylaminopurine, and 0.5 mg L^−1^ of indole‐3‐acetic acid at 25 °C in the dark.

*Nicotiana benthamiana* plants for protein subcellular localization and protein–protein interaction assays were grown in Cornell Mix medium at one plant per pot (10 × 10 × 10 cm) in a controlled growth chamber at 24 °C with 40% to 65% relative humidity under a 16 h photoperiod.

### Transformation of “Royal Gala” Apple with *cMa1*‐OE Vector

The coding sequence (CDS) of *Ma1* (*ALMT9*) from “Usterapfel” high‐acid genotype (*Ma1ma1*) apple^[^ ^12^ ^]^ was cloned into pDONR221 via BP reaction and then transferred into pGWB402 via LR reaction. The resulting *cMa1*‐OE vector was transformed into *A. tumefaciens* strain EHA105 with an additional virulence plasmid pCH32.^[^ ^40^ ^]^ The “Royal Gala” apple was transformed as described.^[^ ^41^ ^]^

### RNA Isolation and Real‐Time Quantitative PCR (RT‐qPCR)

Total RNA was extracted from apple tissues using the modified CTAB method as described previously.^[^ ^42^ ^]^ DNase I enzyme (Thermo Fisher Scientific, USA) was used to remove DNA from 2 µg of total isolated RNA, which was reverse‐transcribed to cDNA using the iScript cDNA Synthesis Kit (Bio‐Rad, USA). RT‐qPCR analysis was performed using SYBR Green Supermix in an iCycler iQ5 system (Bio‐Rad, Hercules, CA, USA) in triplicates, with *MdActin* as the internal reference gene. The relative expression of each gene was calculated using the 2^−ΔΔCT^ method.^[^ ^43^ ^]^ Primers used in this study are listed in Table S3 (Supporting Information).

### Measurements of Malate, Fruit Acidity, and Total Soluble Solids

Fruit samples were taken at 16, 31, 60, 88, and 128 days after bloom (DAB), corresponding to five key developmental stages: active cell division, end of cell division, early rapid cell expansion, late rapid cell expansion, and fruit maturity.^[^ ^2^ ^]^ On each sampling date between noon and 2:00 PM, six well‐exposed fruits were collected from two trees per replicate, immediately cut into pieces after removing the core frozen in liquid nitrogen, and then stored at −80 °C. To measure malate in apple fruit, polar metabolites were extracted in 75% methanol from ≈100 mg of pulverized frozen fruit. Ribitol (0.12 mg per sample) was added to each sample as an internal standard. 10 µL of the aqueous phase were dried under a vacuum without heat after the fractionation of nonpolar metabolites into chloroform. The dried sample was derivatized with methoxyamine hydrochloride and N‐methyl‐N‐trimethylsilyl‐trifluoroacetamide sequentially for analysis on an Agilent 7890A GC/5975C MS (Agilent Technology, Palo Alto, CA, USA) as previously described.^[^ ^44^ ^]^

Fruit titratable acidity (TA) of *cMa1*‐OE transgenic fruits was measured at harvest in two consecutive growing seasons (2018 and 2019). Six fruits pooled from two trees were used for each replicate. Fruit juice TA was quantified with an autotitrator (Metrohm 848 Titrino Plus and Metrohm 869 Compact Sample Changer, Herisau, Switzerland). Total soluble solids were measured with a PAL‐1 digital refractometer (ATAGO, USA).

### Transient Expression of *Ma1α* and *Ma1β* in *Xenopus laevis* Oocytes and Two‐Electrode Voltage Clamp (TEVC) Analysis

The coding sequence of *Ma1α* and *Ma1β* were amplified and cloned into *Xenopus* oocyte expression vectors (with or without N‐terminus YFP, nYFP, or cYFP) by advanced uracil excision‐based cloning technique as previously described by Nour‐Eldin et al.^[^ ^45^ ^]^ cRNA was synthesized using the mMessage mMachine in vitro transcription kit (Invitrogen, USA) and injected into *X. laevis* oocytes as described previously.^[^ ^12^ ^]^ All animal procedures were performed in accordance with Cornell University IACUC Protocol number 2017–0139.

The detection of Ma1α or Ma1β YFP chimeric proteins in *X. laevis* oocytes was carried out on a confocal laser‐scanning microscope (SP5, Leica). The plasma membrane stain (CellMask Plasma Membrane Stains, Deep Red C10064, Thermo Fisher Scientific, USA) was used as a marker for co‐localization of the proteins at the plasma membrane. YFP and Deep Red were excited at 514 and 649 nm, and their emission signals were detected at 520 to 540 nm and 650 to 700 nm, respectively.

Ma1's malate transport activity was recorded via TEVC as described previously.^[^ ^12^ ^]^ Oocytes were injected with 50 nL of water (control) or 50 nL of water solution containing varying amounts of *Ma1α* cRNA and/or *Ma1β* cRNA. Steady‐state current‐voltage (I/V) relationships were constructed by measuring the current amplitude at the end of the test pulse. To detect the effect of Ma1β on Ma1α transporter activity, different amounts of *Ma1β* cRNA were co‐injected with a fixed amount (25 ng) of *Ma1α* cRNA into *X. leavis* oocytes. To determine the response of malate transport activity to the amount of Ma1α co‐expressed with Ma1β, different amounts of *Ma1α* cRNA were co‐injected with *Ma1β* cRNA with their ratio kept constant at 1:1/8.

### Subcellular Localization of Ma1α and Ma1β

cDNAs of *Ma1α* and *Ma1β* were cloned into pGWB551 vector with a C‐terminal GFP via the gateway recombination system (Invitrogen, USA), and were transiently overexpressed in *N. benthamiana* leaves via agroinfiltration as described previously.^[^ ^12^ ^]^ After 2 to 3 days following infiltration, protoplasts were extracted to determine the subcellular localization of Ma1α and Ma1β. The hypotonic medium (10 mm EGTA, 10 mm HEPES/Tris pH 7.4 adjusted to 200 mOsmol kg^−1^ with sorbitol) was applied to release vacuoles via bursting isolated protoplasts. The Ma1α‐ or Ma1β‐GFP signal and chlorophyll auto‐fluorescence were examined using a confocal laser‐scanning microscope (LSM 710; Carl Zeiss) at excitation wavelengths of 488 nm, and the emission signal was detected between 500 and 530 nm for GFP and 650 to 750 nm for chlorophyll auto‐fluorescence. Gateway primers used in this study are listed in Table S3 (Supporting Information).

### Transformation of “Orin” Apple Calli

Transformation of “Orin” apple calli was performed as previously described with slight modifications.^[^ ^12^ ^]^ “Orin” apple calli were cultivated in liquid medium (MS + 1 mg L^−1^ 2,4‐D + 1 mg L^−1^ 6‐BA + 30 g L^−1^ sucrose, pH 5.8–6.0) for two weeks before transformation. The collected calli were incubated with *A. tumefacens* GV3101, harboring various constructs in a liquid medium with shaking at 140 rpm at 25 °C for 15–20 min. The calli were then collected and cultured on solid MS medium (liquid medium with 8 g L^−1^ agarose) at 25 °C. After 3 days, the transgenic calli were washed three times with sterile water and plated on solid selection MS medium with 30 mg L^−1^ kanamycin and 250 mg L^−1^ cefotaxime. The transformed calli were transferred onto a new selection medium every 3 weeks, and the positive transgenic calli were selected via PCR for future analysis.

### Protein Extraction and Immunoblot Analysis

Proteins were extracted from apple tissues and quantified as previously described.^[^ ^46^ ^]^ The primary antibody (anti‐Ma1 antibody, generated in rabbit by Genecript, Co., Ltd, Nanjing, China) was diluted 2000 times, and the secondary antibody (goat anti‐rabbit, alkaline phosphatase‐conjugated, A3687, Sigma, USA) was diluted 5000 times with blocking buffer in TBS (SuperBlock, 37535, Thermo Fisher Scientific, USA) for immunoblot analysis. Then, the 1‐Step NBT/BCIP (1‐Step NBT/BCIP, 34042, Thermo Fisher Scientific, USA) was added to the blot for 5–15 min in the dark until the desired color developed. The SDS‐PAGE gel was stained with a Coomassie Brilliant Blue R‐250 solution (161‐0463, Bio‐Rad, USA) and used to confirm equal loading of proteins.

### Co‐Immunoprecipitation (Co‐IP) Assay

The coding sequences of *Ma1α* and *Ma1β* were both cloned into pGWB417 (C‐terminal fusion with 4xMyc tag) and pGWB414 (C‐terminal fusion with 3xHA tag) via the Gateway recombination system (BP and LR reactions, Invitrogen). The pGWB417‐*Ma1α* or pGWB417‐*Ma1β* in combination with pGWB414‐*Ma1α*, pGWB414‐*Ma1,β* or pGWB414 empty vector were transiently transformed into in *N. benthamiana* leaves as described above and used for Co‐IP assay. The Co‐IP assays were performed following the manufacturer's protocol (Pierce Co‐Immunoprecipitation Kit, 26149, Thermo, USA). Briefly, 1 mg of freshly extracted total protein from agro‐infiltrated *N. benthamiana* leaves was pre‐cleaned with 200 µL agarose resin slurry (2 h, 4 °C). The agarose resin was centrifuged, and the supernatant was transferred into a fresh tube and incubated with anti‐HA/MYC antibody at 4 °C overnight. After brief centrifugation, the sample underwent four washing steps, after which elution buffer was added to obtain the co‐immunoprecipitated proteins for immunoblotting analysis. Primers used in this study are listed in Table S3 (Supporting Information).

### Luciferase (LUC) Complementation Imaging Assay

LUC complementation imaging (LCI) assay was conducted as previously described.^[^ ^47^ ^]^ *Ma1α* and *Ma1β* were both cloned in‐frame to the C‐ and N‐terminal of the LUC reporter in JW772 or JW771 (Primers listed in Table S3, Supporting Information). The resulting constructs were transformed into *A. tumefaciens* GV3101 strain harboring pSoup helper plasmid and then agro‐infiltrated into *N. benthamiana* plants. The infiltrated leaves were sprayed with 1 mm D‐luciferin after 36–48 h, and LUC fluorescence was observed using an in vivo imaging system (NightSHADE L985; Berthold).

### Bimolecular Fluorescence Complementation (BiFC) Assay

The coding sequences of *Ma1α* and *Ma1β* were cloned into the plant binary vectors pSPYNE and pSPYCE to generate nYFP and cYFP fusion proteins.^[^ ^48^ ^]^ Meanwhile, the gene *AtALS3*, encoding a vacuolar membrane protein,^[^ ^29^ ^]^ was cloned into the pSPYCE vector as a negative control for BiFC assay. The constructs were introduced into *A. tumefaciens* GV3101 for infiltration into *N. benthamiana* leaves. At 48 h after agroinfiltration, protoplasts were isolated from the leaves and lysed to release the vacuoles as described for the subcellular localization of Ma1α and Ma1β proteins. BiFC signals were imaged using a Zeiss LSM710 confocal microscope. YFP was excited at 514 nm, and the emission signal was detected at 520 to 540 nm. Gateway primers used in this study are listed in Table S3 (Supporting Information).

### Single‐Molecule Subunit Counting Microscopy

Total internal reflection fluorescence (TIRF) analysis of neoGREEN quenching was performed as described previously with some modifications.^[^ ^30^ ^]^ Briefly, cRNA‐injected *X. leavis* oocytes were incubated for 16–24 h in ND96 working solution at 18 °C. For TIRF imaging, the vitelline membrane was removed manually, and the oocyte was placed on an oxygen plasma cleaned (Basic Plasma Cleaner, Harrick Plasma) glass‐bottom dishes (MatTek Corporation) containing ND96 solution. Single‐molecule imaging was performed on Ma1α‐NeoGREEN using a custom‐built azimuthal scanning objective‐TIRF microscope based on an inverted microscope body (IX‐81, Olympus) with a Flat‐Top XYZ automated stage (Applied Scientific Instrumentation), as described elsewhere.^[^ ^30a^ ^]^ Stepwise photobleaching data were analyzed using a custom lab software package (ImageC.exe, written in C/C++ under Microsoft Visual Studio 2017) which identified fluorescent puncta and recorded the intensity versus time trace.

### Construction of Overexpression and RNAi Viral Vectors and Transient Expression in Apple Fruit

Viral vectors were used for transient gene overexpression or suppression in apple fruit. For overexpression, the CDS of *Ma1α*, *Ma1,β* or *MdMYB73* was cloned into the IL602 vector downstream of the 35S promoter.^[^ ^31e^ ^]^ The plasmid of IL602‐*Ma1α*, *Ma1β*, *MdMYB73* or empty vector IL602 was mixed with help vector IL601 at 1:1 ratio in the injection buffer. ≈200 ng of mixed plasmid DNA (in 100 µL injection buffer) was infiltrated into each site of mature “Royal Gala” apple fruit (three sites per fruit) using a 1‐mL needleless syringe.

For RNAi, a 200 bp fragment of the CDS region shared by *Ma1α* and *Ma1β*, or *MdMYB73* CDS was cloned into the tobacco rattle virus (TRV2) vector in the antisense orientation under the control of the dual 35S promoter.^[^ ^31d^ ^]^ The construct TRV2‐*Ma1*, TRV2‐*MdMYB73*, TRV2 empty vector and helper vector TRV1 were transformed into *A. tumefaciens* GV3101, respectively. Agroinfiltration of mature “Royal Gala” apple fruit was performed as described.^[^ ^12^ ^]^

The infiltrated fruits were kept at room temperature in the dark overnight and then placed into a 16 °C incubator with lights. Each treatment was replicated five times with three fruits per replicate in a completely randomized design. 2 weeks later, the injection regions of the fruit (three sites per fruit) were taken for gene expression and malic acid analysis. Primers used for IL602 or TRV2 constructs are listed in Table S3 (Supporting Information).

### Yeast One‐Hybrid (Y1H) Assay

Y1H assay was performed using yeast (Saccharomyces cerevisiae) strain Y187 (Clontech, USA) according to the manufacturer's instructions. The *MdMYB73* gene was cloned into the pGADT7 vector to generate pGAD‐*MdMYB73*. The promoter fragment of *Ma1* (*proMa1*) was inserted into the pHis2 vector. pHis2‐*proMa1* or pHis2 empty vector was co‐transformed with pGAD‐*MdMYB73* vector into yeast Y187, and the interactions were examined on a medium lacking Leu, Trp, and His (SD/‐Leu‐Trp‐His) with an optimal concentration (50 mm) of 3‐AT over a series of yeast concentrations (10^0^ to 10^−4^). Primers are listed in Table S3 (Supporting Information).

### Chromatin Immunoprecipitation PCR (ChIP‐PCR) Assay

The coding sequence of *MdMYB73* was cloned into the pGWB451 vector, which has a C‐terminal fusion GFP tag. The pGWB451‐*MdMYB73*‐*GFP* and pGWB451 empty vectors were then transformed into apple calli. The positive transgenic calli were selected via PCR and used for ChIP‐PCR assay. ChIP‐PCR was performed according to the protocol of Pierce Agarose ChIP Kit (Thermo; catalog no. 26156). ≈1 g of *MdMYB73‐GFP*‐OE or *GFP*‐OE transgenic calli was cross‐linked in 1% formaldehyde. Subsequently, the immunoprecipitate was used to isolate the protein–DNA complex with a GFP antibody (Invitrogen; product no. PA‐980A, lot no. RH236759). The DNA was purified from the protein–DNA complex using the ChIP kit, and the abundance of each DNA fragment was quantified via qPCR. Primers are listed in Table S3 (Supporting Information).

### Dual LUC Assay

The CDS of *MYB73* was inserted into pGreenII 62‐SK to generate the SK‐MYB73 effector, while the *Ma1* promoter was cloned into the pGreenII0800‐LUC vector as a reporter (Primers listed in Table S3, Supporting Information). *N. benthamiana* leaves were co‐injected with *A. tumefaciens* GV3101 containing both effector and reporter. After 3–5 days, luciferase activities were assessed using the Dual‐Luciferase® Reporter Assay Kit (E1910, Promega, WI, USA) according to the manufacturer's instructions. LUC images were captured with a low‐light cooled CCD imaging system (NightShade LB 985, Berthold, Bad Wildbad, Germany) and analyzed using Indigo software. The relative fluorescence intensity was quantified using an Infinite 200 Pro microplate reader (Tecan, Männedorf, Switzerland). Each combination was replicated three times and the experiment was repeated three times.

### Structural Protein Models of Ma1α and Ma1β

Protein structure models of Ma1α and Ma1β were generated with SWISS‐MODEL using Arabidopsis ALMT1 (PDB‐ID: 7VQ3) as a template.^[^ ^49^ ^]^ Ma1α and Ma1β had QMEANDisCo Global values of 0.60 ± 0.05 and 0.54 ± 0.05, with 87.6% and 88.8% of their residues in the most favored regions in the Ramachandran plot, respectively.^[^ ^50^ ^]^ The Ma1α‐Ma1β heterodimer was modeled using AlphaFold via the Google Colaboratory notebook AlphaFold2.ipynb with the alphafold2_multimer_v3 model for complex prediction, with 20 recycles, an early stop tolerance of 0.5, and relaxation of the best‐ranked model.^[^ ^51^ ^]^ The heterodimer model had a confidence of 0.589, calculated as 0.8× ipTM + 0.2 × pTM8, with an ipTM score of 0.58 and a pTM score of 0.611. Structure representations were generated with the molecular visualization program VMD.^[^ ^52^ ^]^

### Statistical Analysis

Student's *t*‐test or analysis of variance (ANOVA) followed by Tukey's Honestly Significant Difference (HSD) tests were conducted using the software SigmaPlot 11.0 (Systat Software Inc., San Jose, CA, USA).

### Accession Numbers

*MdALMT9* (*Ma1*) (MDP0000252114, MD16G1045200), *MdMYB73* (MDP0000894463, MD08G1107400), *AtALS3* (AT2G37330)

## Conflict of Interest

The authors declare no conflict of interest.

## Author Contributions

L.C., M.A.P., K.X., and C.L. conceived and designed the study. C.L., S.K., M.Z., D.H., D.M., and L.D. conducted the experiments and data analysis. J.R. and M.A.P. did protein homology modeling. C.L., L.C., and M.A.P. wrote the manuscript with inputs from all other authors. L.C. is responsible for the distribution of materials integral to the findings presented in this article.

## Supporting information

Supporting Information

## Data Availability

The data that support the findings of this study are available from the corresponding author upon reasonable request.
