# Supplementary material for: Alternative Splicing Underpins the ALMT9 Transporter Function for Vacuolar Malic Acid Accumulation in Apple
Source: Adv Sci (Weinh). 2024 Mar 21;11(22):2310159. doi: 10.1002/advs.202310159 (PMC11165477; doi:10.1002/advs.202310159)
Supplement: Supplementary file 1 — Supporting Information [file ADVS-11-2310159-s001.pdf]

ADVANCED SCIENCE
Open Access

Supporting Information

for *Adv. Sci.*, DOI 10.1002/advs.202310159

Alternative Splicing Underpins the ALMT9 Transporter Function for Vacuolar Malic Acid Accumulation in Apple

Chunlong Li, Srinivasan Krishnan, Mengxia Zhang, Dagang Hu, Dong Meng, Janin Riedelsberger, Laura Dougherty, Kenong Xu, Miguel A. Piñeros and Lailiang Cheng**

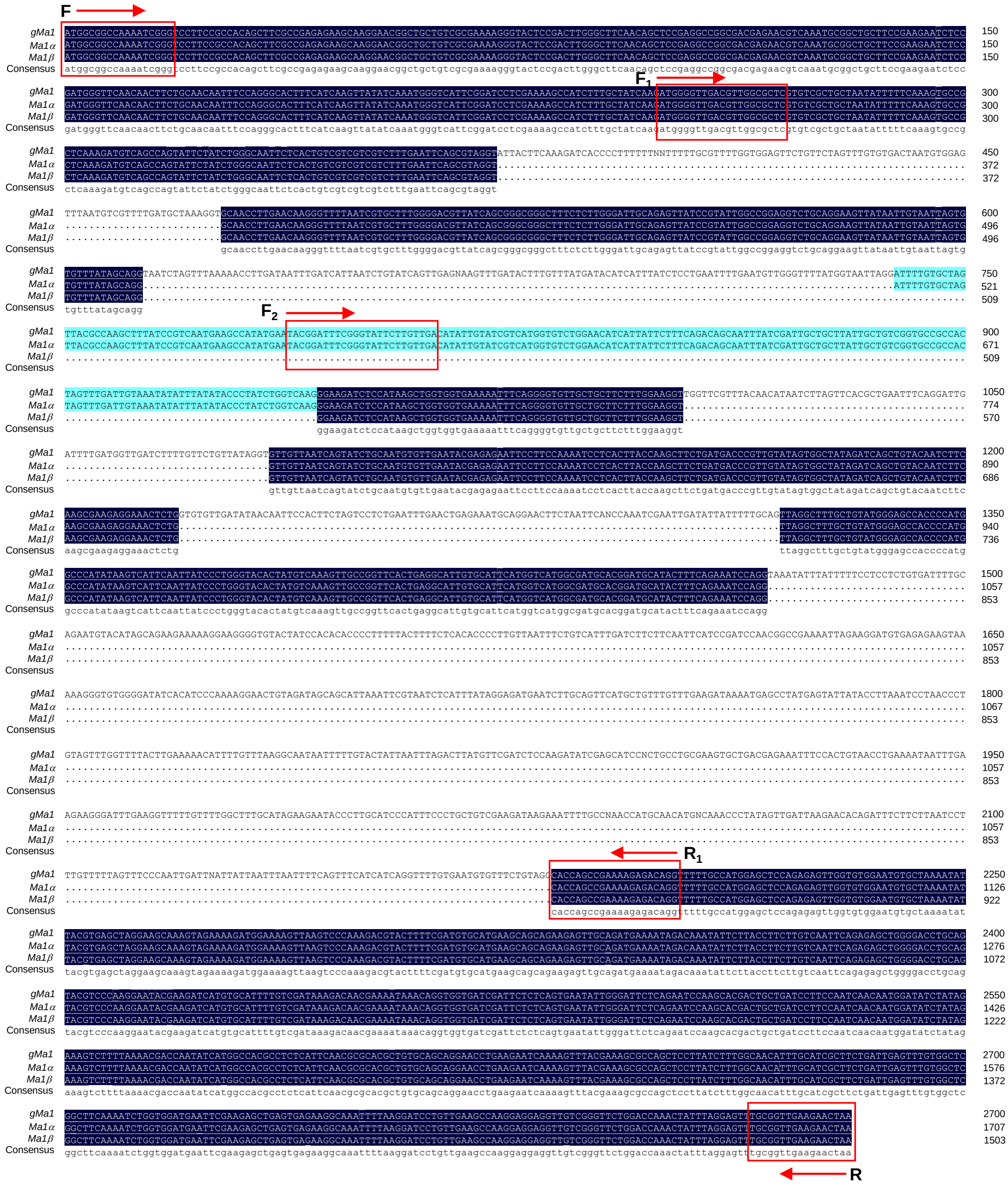

Supplemental Figure 1. Nucleotide sequence alignment of genomic *Ma1* (*gMa1*), *Ma1α* CDS and *Ma1β* CDS using DNAMAN software.

The primers used for the PCR assay of Figure 2A-C are highlighted in the red box.

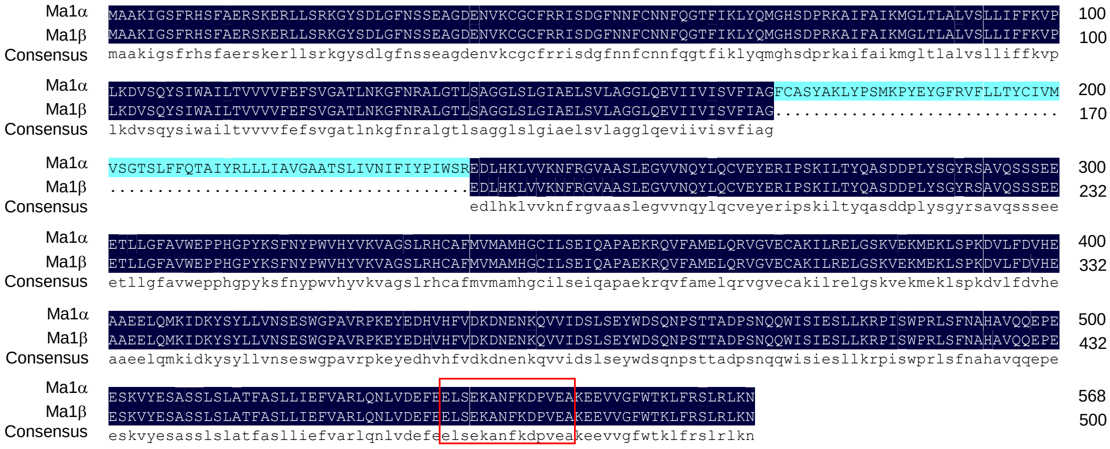

Supplemental Figure 2. Protein sequence alignment of Ma1α and Ma1β using DNAMAN software.

The peptide sequence ELSEKANFKDPVEA used to generate the antibody against Ma1 is highlighted in the red box.

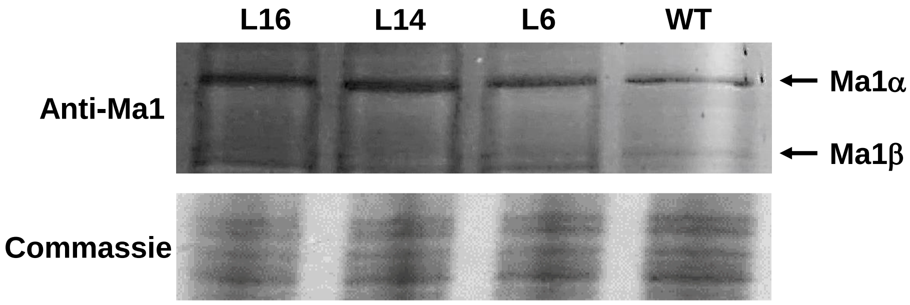

Supplemental Figure 3. Detection of Ma1α and Ma1β proteins by immunoblotting at the peak of malic acid accumulation (31 days after bloom) during fruit development in wild type (WT) and *cMa1*-OE lines (L6, L14, L16) of 'Royal Gala' apple.

The antibody was generated against peptide ELSEKANFKDPVEA in rabbit, which recognizes both Ma1α and Ma1β.

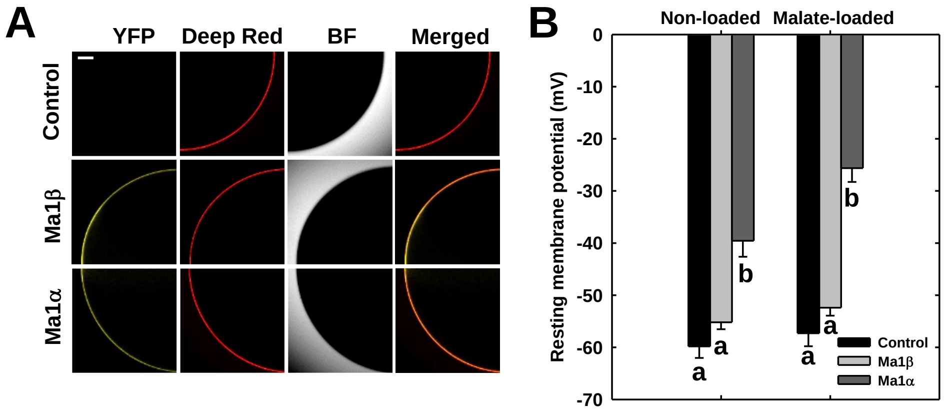

Supplemental Figure 4. Localization of Ma1β-YFP and Ma1α-YFP fusion proteins in oocytes and Resting Membrane Potentials (RMPs) recorded in control and *Ma1β* or *Ma1α*-expressing cells

(A) Expression and localization of Ma1β-YFP and Ma1α-YFP fusion proteins in oocytes, with Deep Red as a plasma membrane marker (PM Marker). Each oocyte was injected with 25 ng cRNA. Bar = 100 µm. BF: Bright Field.

(B) RMPs recorded in control and Ma1β or Ma1α-expressing cells. RMPs were recorded in cells non-loaded or loaded with malate by microinjecting cells with 50 nl of 100 mM Na-Malate (increasing cytosolic malate^{2-} concentration by 4.5 mM) 2 to 3 hours prior to the electrophysiological recordings. Data are mean ± SE. The number of cells recorded: Control-non-loaded (n = 11), Ma1β-non-loaded (n = 18), Ma1α-non-loaded (n = 9), Control-malate-loaded (n = 10), Ma1β-malate-loaded (n = 20), and Ma1α-malate-loaded (n = 19). Different letters (a, b, c) indicate significant differences between groups using Tukey's HSD test at $P < 0.05$ after ANOVA.

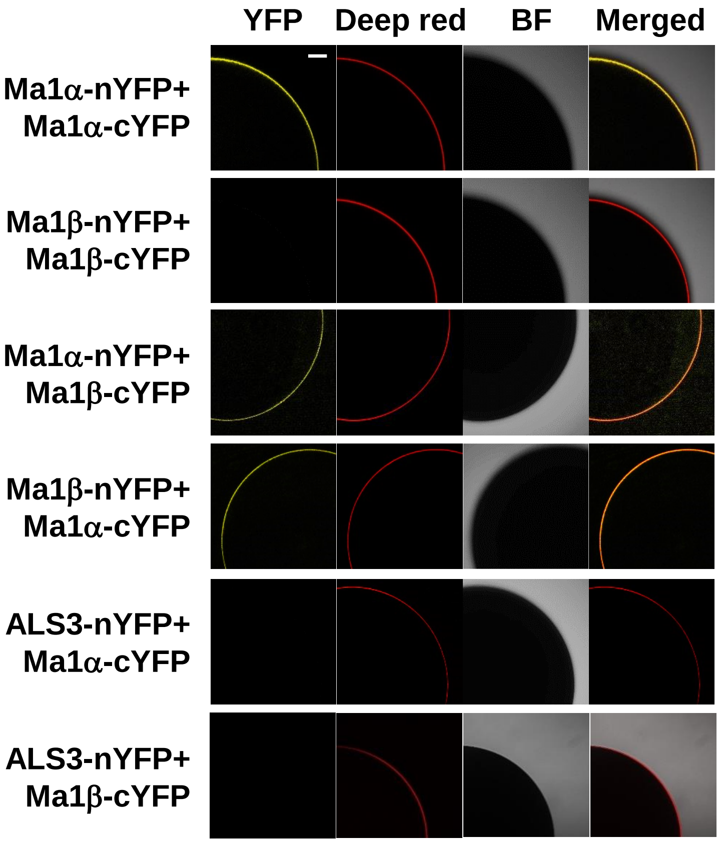

Supplemental Figure 5. BiFC assays of Ma1α and Ma1β in *Xenopus laevis* oocytes.

BiFC assays of Ma1α and Ma1β in *X. laevis* oocytes co-expressing Ma1α-nYFP/Ma1α-cYFP, Ma1β-nYFP/Ma1β-cYFP, Ma1α-nYFP/Ma1β-cYFP, or Ma1β-nYFP/Ma1α-cYFP fusion constructs, with a tonoplast localized protein ALS3 and empty vector (nYFP) as negative controls. Deep Red was used as a plasma membrane marker (PM Marker). Bar = 100 μm. BF: Bright Field.

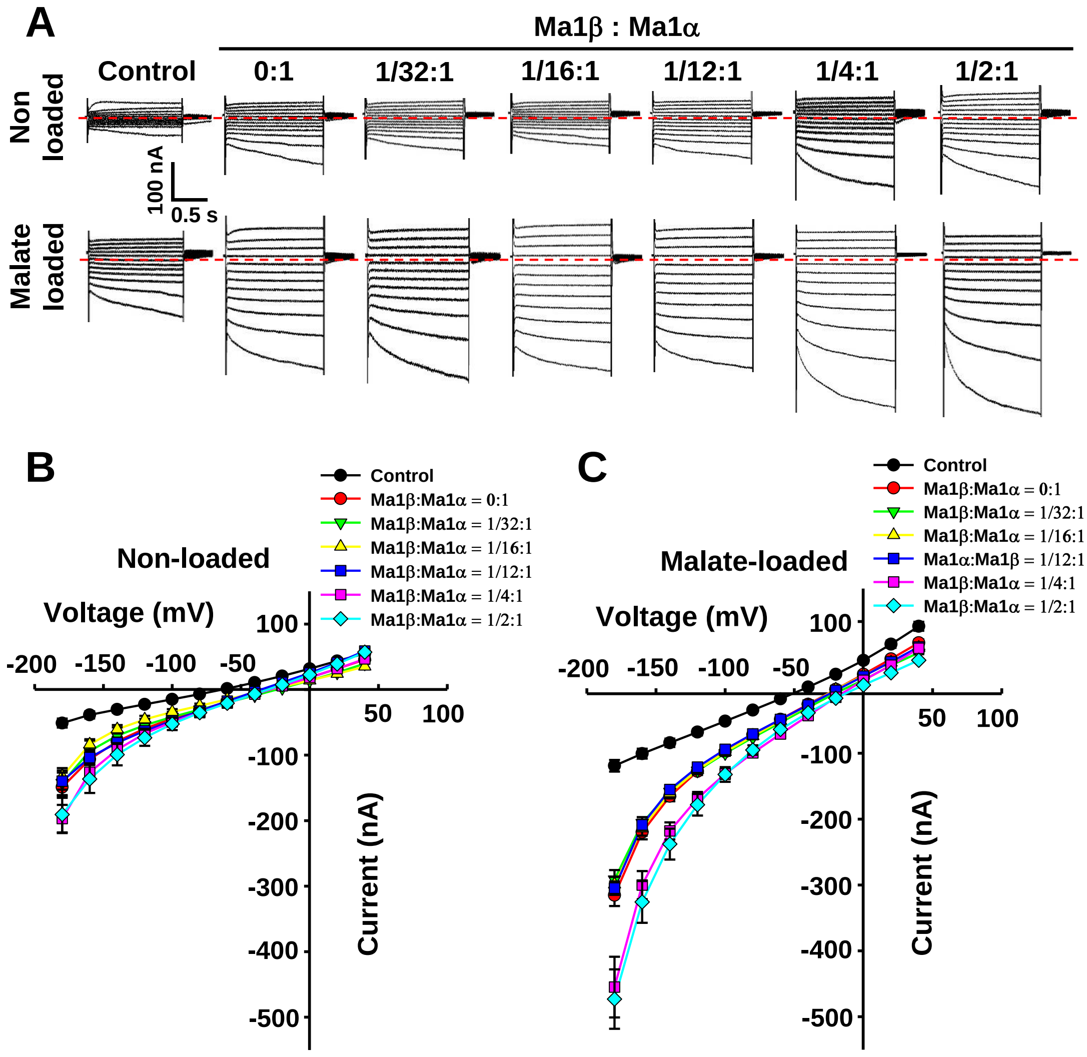

Supplemental Figure 6. Malate transport activity in *Xenopus laevis* oocytes co-expressing *Ma1α* and *Ma1β* genes in various ratios.

(A) Examples of currents elicited in response to holding potentials ranging from +40 to -180 mV (in 20 mV steps) recorded in control, *Ma1β* / *Ma1α* co-expressing cells, either non-loaded or loaded with malate. Ma1β: Ma1α = 0:1, 1/32:1, 1/16:1, 1/12:1, 1/4:1, 1/2:1 at a fixed amount (25 ng) of Ma1α cRNA. The zero-current level is indicated by the red dotted line.

(B) Current-voltage (I/V) relationships constructed from steady-state current recordings with non-loaded cells such as those shown in **A**. Data are mean ± SE. The number of cells recorded: Control (n = 18), 0:1 (n = 15), 1/32:1 (n = 10), 1/16:1 (n = 10), 1/12:1 (n = 10), 1/4:1 (n = 10), and 1/2: 1 (n = 10).

(C) Current-voltage (I/V) relationships constructed from steady-state current recordings with malate-loaded cells such as those shown in **A**. Data are mean ± SE. The number of cells recorded: Control (n = 16), 0:1 (n = 17), 1/32:1 (n = 10), 1/16:1 (n = 24), 1/12:1 (n = 22), 1/4:1 (n = 22), and 1/2:1 (n = 15).

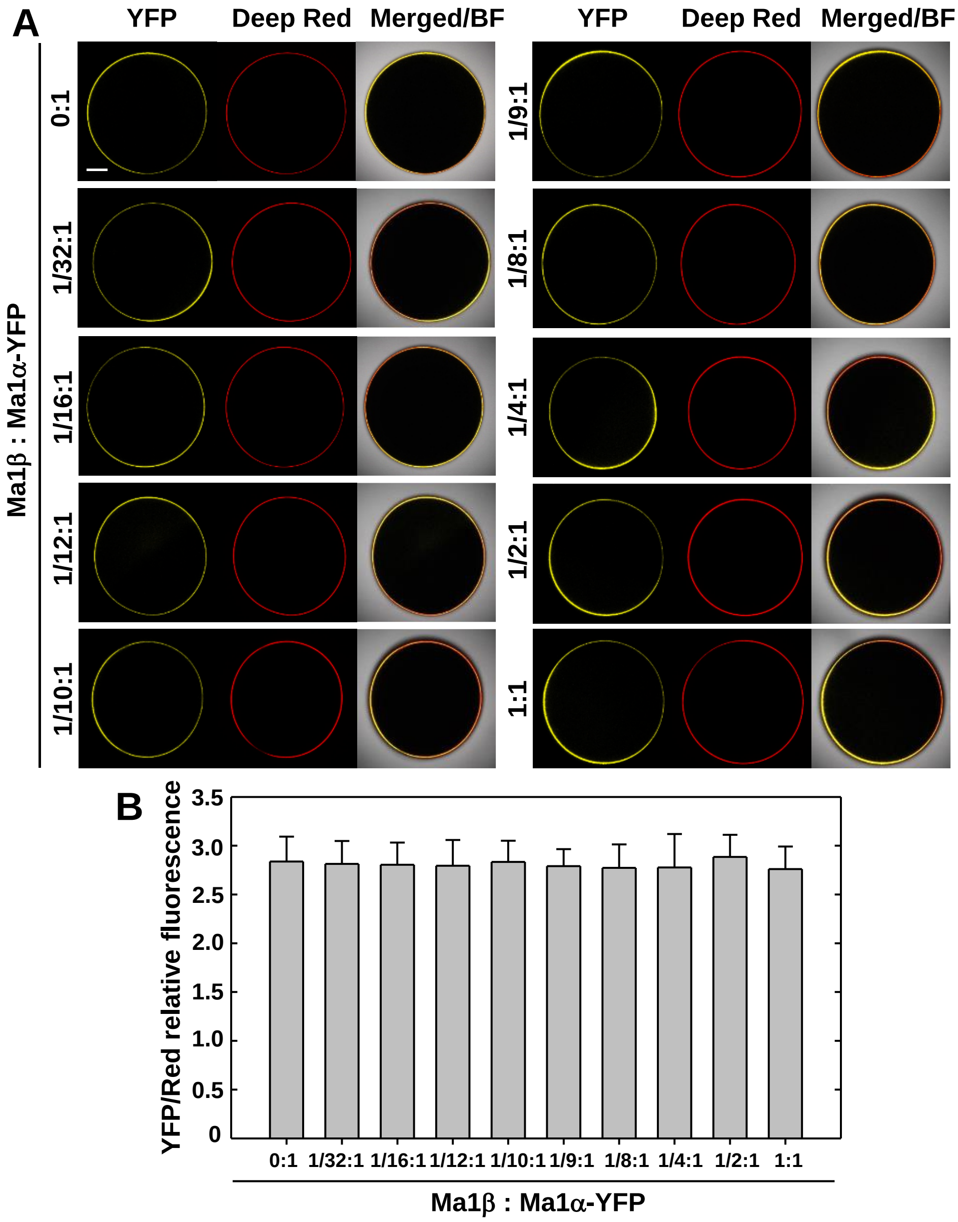

Supplemental Figure 7. Ma1α protein expression is not affected by injection of *Ma1β* cRNA at different ratios in *Xenopus laevis* oocytes.

(A) Representative images of the Ma1α-YFP fluorescence signals in oocytes co-injected with *Ma1β* at the indicated ratios, at a fixed amount of Ma1α cRNA (25 ng) per oocyte. Deep red was used as a plasma membrane marker and a reference fluorescence signal. Bar = 200 μm. BF: Bright Field.

(B) The expression of Ma1α protein indicated by the YFP/Red relative fluorescence in response to the addition of *Ma1β*. Data are mean ± SE of 20 cells recorded.

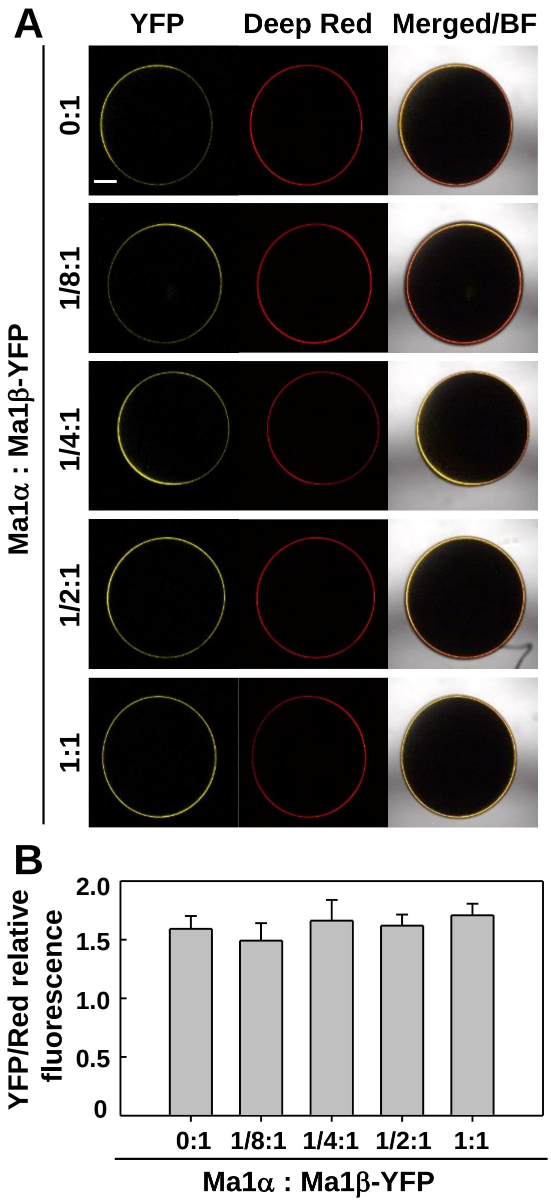

Supplemental Figure 8. Ma1β protein expression is not affected by co-injection of *Ma1α* cRNA at different ratios in *Xenopus laevis* oocytes.

(A) Representative images of the Ma1β-YFP fluorescence signals in oocytes co-injected with *Ma1α* at the indicated ratios, at a fixed amount of Ma1β cRNA (25 ng) per oocyte. Deep red was used as a plasma membrane marker and a reference fluorescence signal. Bar = 200 μm. BF: Bright Field.

(B) The expression of Ma1β protein indicated by the YFP/Red relative fluorescence in response to the addition of *Ma1α*. Data are mean ± SE of 20 cells recorded.

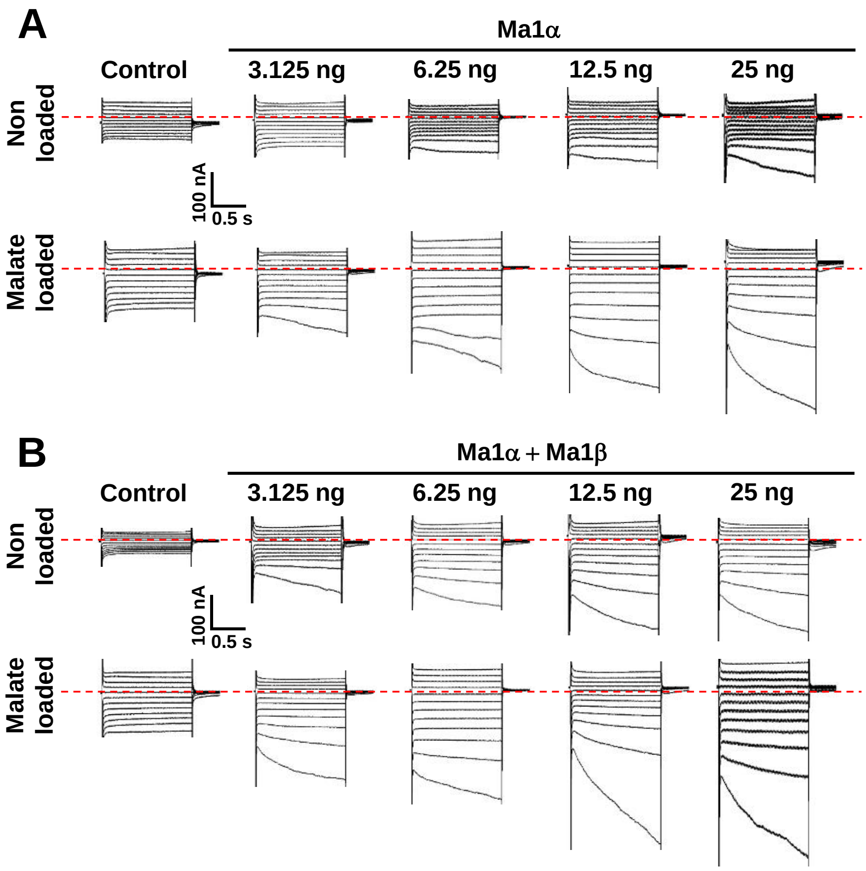

Supplemental Figure 9. Traces of currents elicited in *Xenopus laevis* oocytes in response to increasing amounts of Ma1α cRNA alone or co-expressed at a constant ratio with *Ma1β*.

(A) Examples of currents elicited in response to holding potentials ranging from +40 to -180 mV (in 20 mV steps) recorded in non-loaded and malate-loaded oocyte cells injected with various amounts of *Ma1α* cRNA (0 to 25 ng). The red dotted line indicates the zero-current level.

(B) Examples of currents elicited in response to holding potentials ranging from +40 to -180 mV (in 20 mV steps) recorded in non-loaded and malate-loaded oocyte cells injected with *Ma1α* and *Ma1β* cRNA in a constant ratio of 1 to 1/8 at various amounts of *Ma1α* cRNA (0 to 25 ng). The red dotted line indicates the zero-current level.

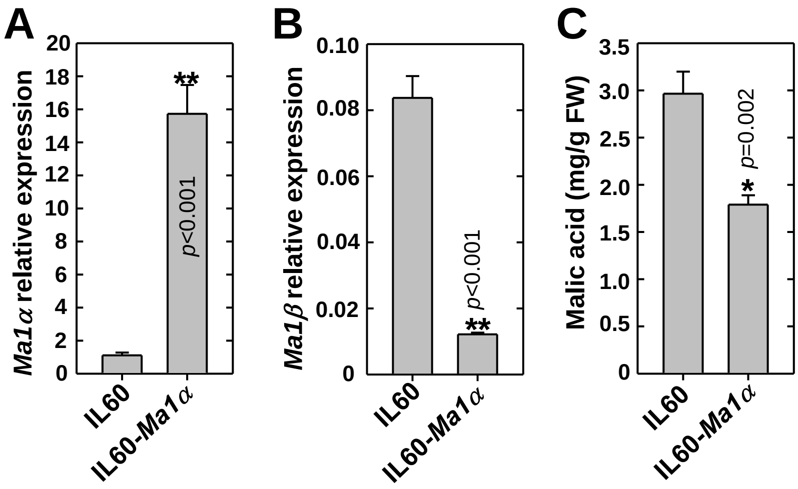

Supplemental Figure 10. Virus-based gene-overexpression of *Ma1α* decreases malic acid concentrations in wild-type 'Royal Gala' mature fruit.

(A) The expression of *Ma1α* after rattle virus-based gene-overexpression of *Ma1α*.

(B) The expression of *Ma1β* after rattle virus-based gene-overexpression of *Ma1α*.

(C) Fruit malic acid concentrations in response to rattle virus-based gene-overexpression of *Ma1α*.

Virus vector IL60-*Ma1α* was infiltrated into fruit for overexpression of *Ma1α*, with empty vector (IL60) as a control. Data are mean ± SE of 5 biological replicates with 3 fruits per replicate (3 injection sites per fruit). * Represents significant differences using Student's *t* test at $P < 0.05$, ** Represents significant differences using Student's *t* test at $P < 0.01$.

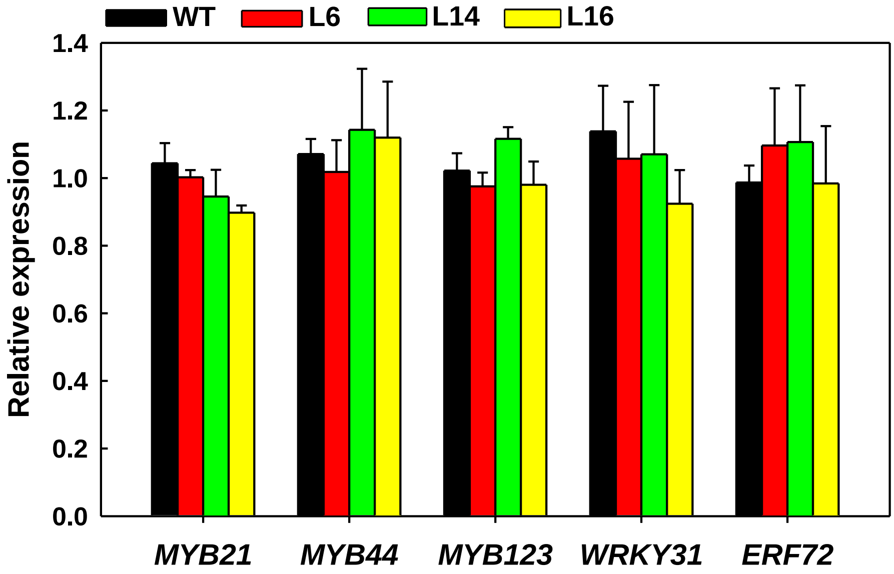

Supplemental Figure 11. Expression levels of several transcription factors at the peak of malic acid accumulation (31 days after bloom) during fruit development in wild type (WT) and *cMa1*-OE lines (L6, L14, L16) of 'Royal Gala' apple.

Quantitative RT-PCR was performed using gene-specific primers (Supplemental Table 1), with *actin* as the internal reference gene, and the relative expression level of each gene was obtained using the ddCT method. Data are mean ± SE of 5 biological replicates, with 6 fruits pooled from 2 trees per replicate.

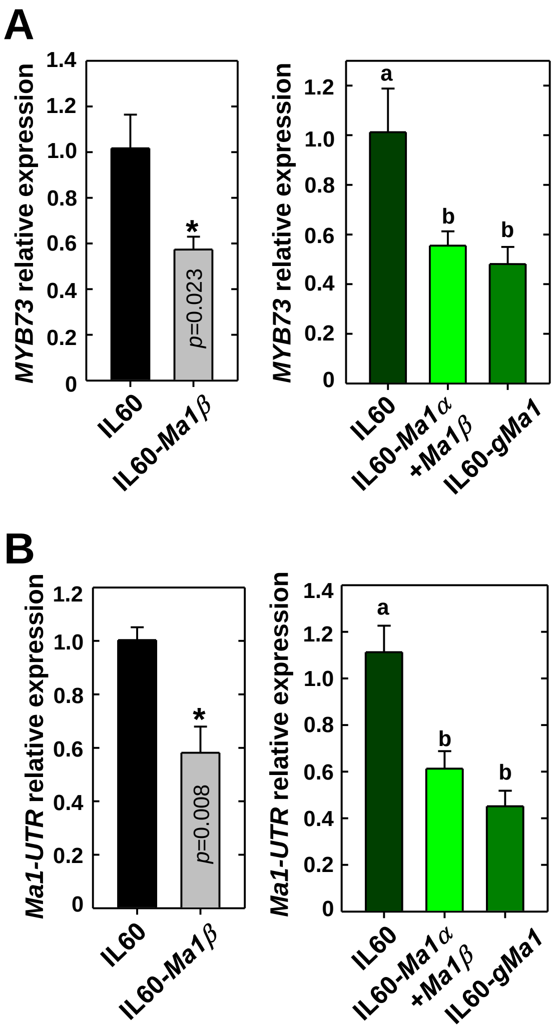

Supplemental Figure 12. Transcript levels of *MYB73* and the native *Ma1* gene in response to overexpression of *Ma1β*, *Ma1α* and *Ma1β*, or *gMa1* in wild-type 'Royal Gala' apple.

(A) The expression level of *MYB73* after rattle virus-based gene-overexpression of *Ma1β*, both *Ma1α* and *Ma1β* or genomic sequence of *Ma1* (*gMa1*) in WT mature fruits. Fruit was infiltrated with virus vector IL60-*Ma1β* for overexpression of *Ma1β*. IL60-*Ma1α* and IL60-*Ma1β* were co-infiltrated, or IL60-*gMa1* was infiltrated into fruits for overexpression of *Ma1α* and *Ma1β*. Fruit infiltrated with empty vector (IL60) was used as control.

(B) The expression level of native *Ma1* gene after rattle virus-based gene-overexpression of *Ma1β*, both *Ma1α* and *Ma1β* or genomic sequence of *Ma1* (*gMa1*) in WT mature fruits. Fruit was infiltrated with virus vector IL60-*Ma1β* for overexpression of *Ma1β*. IL60-*Ma1α* and IL60-*Ma1β* were co-infiltrated, or IL60-*gMa1* was infiltrated into fruits for overexpression of *Ma1α* and *Ma1β*. Fruit infiltrated with empty vector (IL60) was used as control.

Quantitative RT-PCR was performed using gene-specific primers (Supplemental Table 1), with *actin* as the internal reference gene, and the relative expression level of each gene was obtained using the ddCT method. Data are mean ± SE of 5 biological replicates with 3 fruits

per replicate (3 injection sites per fruit). * Represents significant differences using Student's *t* test at P < 0.05. Different letters (a, b) indicate significant difference between groups using Tukey's HSD test at *P* < 0.05 after ANOVA.

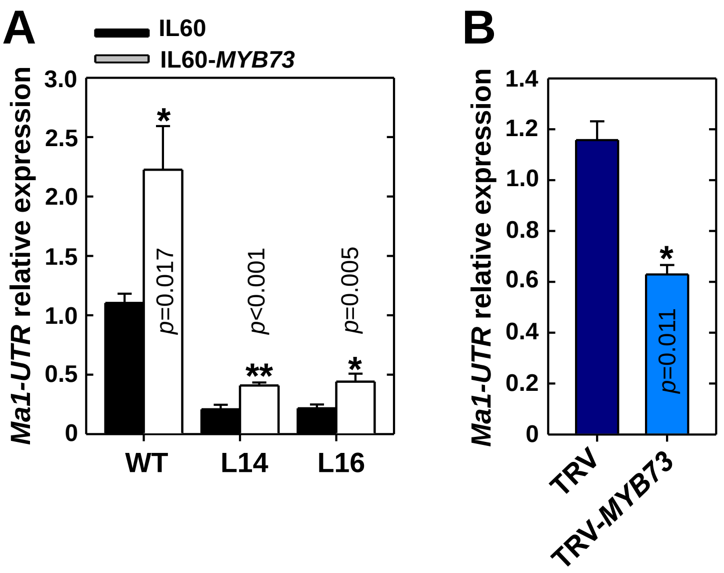

Supplemental Figure 13. The native *Ma1* transcript levels in response to virus-based gene-overexpression or silencing of *MdMYB73* in mature fruits of wild-type and *cMa1*-OE lines of 'Royal Gala' apple.

(A) The expression of native *Ma1* in response to rattle virus-based gene-overexpression of *MdMYB73* in mature fruits of WT and *cMa1*-OE lines (L14 and L16).

(B) The expression of native *Ma1* in response to rattle virus-induced gene-silencing of *MdMYB73* in WT mature fruit.

Virus vectors IL60-*MYB73* and TRV-*MYB73* were infiltrated into fruit for overexpression and silencing of *MYB73*, with empty vectors (IL60 or TRV) as control, respectively. Data are mean ± SE of 5 biological replicates with 3 fruits per replicate (3 injection sites per fruit). * Represents significant differences using Student's *t* test at P < 0.05, ** Represents significant differences using Student's *t* test at P < 0.01.

Supplemental Table 1. Statistical analysis of transmembrane current (nA) at various ratios of Ma1β/Ma1α under malate-loaded conditions in oocytes.

	-180 mV	-160 mV	-140 mV	-120 mV	-100 mV	-80 mV	-60 mV	-40 mV	-20 mV	0 mV	20 mV	40 mV
Control	-112.60±4.64 a	-89.10±4.17 a	-72.26±3.73 a	-57.16±3.21 a	-42.62±2.67 a	-28.19±2.17 a	-13.91±1.75 a	0.68±1.55 a	16.19±1.69 a	33.16±2.26 a	53.26±3.15 a	76.80±3.95 a
Ma1β:Ma1α = 0:1	-300.86±8.23 b	-206.86±6.33 b	-154.49±4.93 b	-118.55±3.91 b	-90.69±3.18 b	-66.87±2.59 b	-45.23±2.11 b	-24.88±1.73 b	-5.11±1.63 b	14.50±2.03 b	34.80±2.83 b	56.31±3.80 b
Ma1β:Ma1α = 1/32:1	-291.76±14.43 b	-205.90±8.96 b	-158.61±6.59 b	-126.41±5.21 b	-99.90±3.75 b	-76.18±2.57 b	-53.61±1.66 b	-30.64±2.16 b	-8.47±2.89 b	11.02±1.99 b	33.39±2.65 b	52.54±2.19 b
Ma1β:Ma1α = 1/16:1	-299.25±11.57 b	-214.14±11.03 b	-160.57±8.02 b	-125.22±6.52 b	-98.08±5.78 b	-73.88±4.97 b	-50.72±4.27 b	-27.22±3.71 b	-3.19±3.33 b	17.88±2.39 b	37.77±2.02 b	60.14±2.03 b
Ma1β:Ma1α = 1/12:1	-304.83±10.14 b	-208.80±7.94 b	-155.19±6.27 b	-121.16±5.08 b	-94.08±4.07 b	-70.78±3.35 b	-48.69±2.96 b	-26.84±2.58 b	-4.69±2.43 b	16.98±2.68 b	38.81±3.19 b	61.26±3.83 b
Ma1β:Ma1α = 1/10:1	-308.66±13.21 b	-219.93±9.68 b	-163.19±6.71 b	-128.11±5.08 b	-100.29±4.13 b	-76.57±3.42 b	-54.83±2.77 b	-33.86±2.18 b	-12.49±1.85 c	9.64±2.07 b	33.19±2.96 b	58.49±4.35 b
Ma1β:Ma1α = 1/9:1	-329.18±18.99 b	-231.59±11.88 b	-172.16±9.53 b	-133.91±7.08 b	-103.68±5.42 b	-77.66±4.19 b	-53.96±3.23 b	-30.95±2.48 b	-8.56±2.03 b	13.48±2.42 b	35.77±3.63 b	59.20±5.36 b
Ma1β:Ma1α = 1/8:1	-454.62±10.30 c	-296.18±7.21 c	-212.51±5.64 c	-160.70±4.20 c	-123.52±3.07 c	-93.38±2.22 c	-67.47±1.60 c	-43.60±1.20 c	-20.55±1.06 c	1.89±1.22 c	24.08±1.61 b	46.62±2.16 b
Ma1β:Ma1α = 1/4:1	-454.96±46.06 c	-300.20±21.36 c	-217.95±13.02 c	-168.72±10.00 c	-131.27±7.69 c	-99.52±6.15 c	-70.84±4.60 c	-43.02±3.30 c	-15.08±3.05 c	10.61±3.92 b	33.55±5.27 b	59.20±6.47 b
Ma1β:Ma1α = 1/2:1	-473.26±45.09 c	-325.74±31.69 c	-237.94±23.49 c	-178.20±16.39 c	-132.23±11.16 c	-95.11±7.24 c	-63.83±4.42 c	-38.56±2.50 c	-16.58±1.49 c	3.48±1.12 c	22.15±1.22 b	41.09±1.87 b
Ma1β:Ma1α = 1:1	-466.60±19.21 c	-314.89±13.73 c	-230.52±11.39 c	-173.52±9.24 c	-131.01±7.27 c	-95.80±5.49 c	-66.61±4.13 c	-41.02±3.05 c	-18.14±2.19 c	4.44±1.80 c	28.12±2.32 b	54.21±3.92 b

Note: Different letters (a, b, c) indicate significant difference between groups using Tukey's Honest Signi fi cant Difference test at $P < 0.05$ after one-way ANOVA.

Supplemental Table 2. Statistical analysis of transmembrane current (nA) at different amounts of Ma1α alone or co-expressed with Ma1β (at 1/8 Ma1α) under malate-loaded conditions in oocytes.

		-180 mV	-160 mV	-140 mV	-120 mV	-100 mV	-80 mV	-60 mV	-40 mV	-20 mV	0 mV	20 mV	40 mV
0 ng Ma1α	Control-1	-103.35±7.74	-84.17±6.13	-69.36±5.19	-54.15±3.97	-37.88±2.93	-21.27±2.17	-4.63±1.66	11.51±1.57	26.57±1.68	41.90±2.24	58.99±3.10	71.36±2.83
	Control-2	-101.59±12.10	-75.78±10.71	-59.47±9.14	-45.32±7.67	-30.68±6.28	-15.74±4.99	-0.82±3.73	13.66±2.59	26.92±1.76	40.13±1.39	56.36±1.87	69.68±2.45
	P vaule	0.904	0.904	0.372	0.334	0.328	0.339	0.379	0.499	0.889	0.503	0.468	0.658
3.125 ng Ma1α	Ma1α	-170.83±10.10	-125.77±7.89	-99.35±6.83	-76.99±5.72	-55.85±4.72	-35.29±3.61	-15.01±2.50	4.62±1.61	22.74±1.32	40.67±1.76	59.29±2.63	78.34±4.12
	Ma1α+Ma1β	-280.47±17.93*	-193.56±17.63*	-149.42±15.52*	-114.37±13.42*	-83.15±10.89*	-54.57±8.25*	-27.78±5.62*	-2.30±3.11*	20.46±1.64	41.28±3.14	62.18±5.71	85.34±8.95
	P vaule	<0.001	0.001	0.004	0.011	0.021	0.030	0.035	0.047	0.287	0.860	0.624	0.451
6.25 ng Ma1α	Ma1α	-235.34±29.88	-150.91±10.12	-111.26±5.76	-84.85±4.80	-61.65±3.89	-40.51±2.91	-19.83±2.13	0.19±1.59	18.19±1.36	35.18±1.78	52.65±2.76	72.05±4.15
	Ma1α+Ma1β	-432.63±35.16*	-255.76±19.55*	-178.87±17.30*	-132.97±14.79*	-97.84±12.35*	-68.23±10.07*	-40.59±7.68*	-14.45±5.34*	9.56±1.95*	30.70±1.72	50.10±2.35	74.24±4.98
	P vaule	<0.001	<0.001	0.003	0.011	0.020	0.027	0.029	0.028	0.003	0.090	0.490	0.747
12.5 ng Ma1α	Ma1α	-340.73±19.45	-197.47±10.15	-130.87±8.05	-92.93±6.35	-64.69±4.96	-41.06±3.80	-20.14±2.84	-1.08±1.98	15.31±1.91	30.48±2.57	46.24±3.67	63.64±5.04
	Ma1α+Ma1β	-526.67±58.45*	-285.59±24.10*	-182.37±12.74*	-125.27±8.76*	-79.63±4.38*	-55.98±1.96*	-29.07±2.67*	-8.66±2.85*	9.99±2.32	26.74±2.07	43.81±2.23	64.84±2.79
	P vaule	0.006	0.003	0.003	0.008	0.039	0.004	0.036	0.041	0.093	0.282	0.590	0.843
25 ng Ma1α	Ma1α	-333.37±34.87	-188.46±16.19	-122.49±9.59	-85.39±6.31	-59.46±4.53	-38.87±3.36	-21.14±2.57	-4.60±1.92	9.73±1.52	24.63±2.17	40.73±5.31	45.69±4.01
	Ma1α+Ma1β	-568.68±69.48*	-311.58±36.61*	-206.52±37.36*	-153.95±21.45*	-117.68±12.38*	-85.20±12.12*	-52.51±12.60*	-22.42±7.96*	8.88±4.41	30.61±2.01	55.37±5.31	65.32±7.46*
	P vaule	0.008	0.007	0.045	0.007	<0.001	0.002	0.027	0.045	0.858	0.061	0.069	0.034

Note: Student *t*-test was used to detect the difference between Ma1α alone and co-expression of Ma1α and Ma1β at each level of Ma1α. An asterisk indicates a significant difference at $P < 0.05$.

Supplemental Table 3. List of primers used in this paper.

Name	Sequence (5'-3')
**Gateway primers**	
Ma1-GW-F	GGGGACAAGTTTGTACAAAAAAGCAGGCTTCATGGCGGCCAAAATCGG
Ma1-GW-R	GGGGACCACTTTGTACAAGAAAGCTGGGTCTTAGTTCTTCAACCGCA
Ma1-GW-R-W/O	GGGGACCACTTTGTACAAGAAAGCTGGGTCGTTCTTCAACCGCAAAC
MYB73-GW-F	GGGGACAAGTTTGTACAAAAAAGCAGGCTTCATGGAAGCGATGAATATGTGC
MYB73-GW-R	GGGGACCACTTTGTACAAGAAAGCTGGGTCTTAATTTAATCTATGAAGCTCCG
MYB73-GW-R-W/O	GGGGACCACTTTGTACAAGAAAGCTGGGTCATTTAATCTATGAAGCTCCGAAG
AtALS3-GW-F	GGGGACAAGTTTGTACAAAAAAGCAGGCTTCATGGATCTGAAATGGGATGAT
AtALS3-GW-R	GGGGACCACTTTGTACAAGAAAGCTGGGTCTCAATCTGAGGAGAAGACATG
**Alternative splicing primers**	
AS-F	ATGGCGGCCAAAATCGG
AS-R	TTAGTTCTTCAACCGCA
AS-F1	ATGGGGTTGACGTTGGCGCTC
AS-R1	CCTGTCTCTTTTCGGCTGGTG
AS-F2	TACGGATTTCGGGTATTCTTGTTGA
**RT-qPCR**	
qMa1-UTR-F	TTCAAATCCTTCTTCTCCGACG
qMa1-UTR-R	CTGTTGAAGCCCAAGTCGGAG
qMYB73-F	CCAACAAGCCCGAACGAA
qMYB73-R	ACCACCTGAGCCTGCACGAC
qMa1β-F	GTGTGTTTATAGCAGGGGAAGATC
qMa1β-R	CAACGGGTCATCAGAAGCTTGG
qMa1α-F	CAGGATTTTGTGCTAGTTACGCC
qMa1α-R	CAGCAATAAGCAGCAATCGATA

qMa1-F	GCGGCTGCTTCCGAAGAATCTC
qMa1-R	TATTAGCAGCGACACGAGCGCC
qRT-MdMYB21-F	CCTTCCGTGCCACTCTCAAC
qRT-MdMYB21-R	TAAGCTGCTGTAGCCAAGTCG
qRT-MdMYB44-F	ATCGCTCAGCCTTTCCCTTC
qRT-MdMYB44-R	TCTCGCTGTTCTGGTTGCTC
qRT-MdMYB123-F	AGGAAACAAATGCAGGGGGA
qRT-MdMYB123-R	AGTCGTCGTGGTTAATCTCCG
qRT-MdWRKY31-F	ACCAACAACTACAGTGCACTGC
qRT-MdWRKY31-R	CAATCCTTGCTTCTTTTCTTGATC
qRT-MdERF72-F	AGAGGGCTCTTTTTTCTC
qRT-MdERF72-R	ACCTTGTGATTCTCCGG
qActin-F	TGACCGAATGAGCAAGGAAATTACT
qActin-R	TACTCAGCTTTGGCAATCCACATC
**User primers**	
Ma1-USER-F	GGCTTAAUATGGCGGCCAAAATCGG
Ma1-USER-R	GGTTTAAUCCTTAGTTCTTCAACCGCAAAC
Ma1-USER-R-W/O	GGTTTAAUCCGTTCTTCAACCGCAAACTCC
**IL60 expression**	
Ma1-IL60-F	CCGTCGACATGGCGGCCAAAATCGG
Ma1-IL60-R	GCTCTAGATTAGTTCTTCAACCGCAAACTCC
MYB73-IL60-F	CCGTCGACATGGAAGCGATGAATATGTGC
MYB73-IL60-R	GCTCTAGATTAATTTAATCTATGAAGCTCCG
**TRV2 expression**	
Ma1-TRV2-F	GCTCTAGAATGGCGGCCAAAATCGG
Ma1-TRV2-R	GGGGTACCAACTTGATGAAAGTGCCCTGG
MYB73-TRV2-F	GCTCTAGAATGGAAGCGATGAATATGTGC
MYB73-TRV2-R	GGGGTACCCGACCTTTTATGTACCGGC

**Y1H assay**	
pGADT7-MdMYB73-F	TGGAATTCATGGAAGCGATGAATATGTGC
pGADT7-MdMYB73-R	TAGGATCCTTAATTTAATCTATGAAGCTCCG
pHis2-Ma1-Pro-F	TAGAATTCGTATAAATCATGTAAGCCAGGTG
pHis2-Ma1-Pro-R	TTGAGCTCTTCGTCGGAGAAGAAGGATTTG
**ChIP-PCR**	
Ma1-ChIP-F	CCAAAATAGTATAACCACATGTCTCC
Ma1-ChIP-R	GAGGGAAAGTGGAAAGCAGAG
**Dual LUC assay**	
MYB73-SK-F	GAATTCTGGAAGCGATGAATATGTG
MYB73-SK-R	GTCGAGATTTAATCTATGAAGCTCCG
Ma1-Pro-LUC-F	GTCGACAGAGAGAGTTATAAGCTTAG
Ma1-Pro-LUC-R	GGATCCTTGTTGGAAGAGGGAAAGTG
**LCI assay**	
Ma1-JW771-F	ACGGGGGACGAGCTCGGTACCATGGCGGCCAAAATCGG
Ma1-JW771-R	CGCGTACGAGATCTGGTCGACGTTCTTCAACCGCAAAC
Ma1-JW772-F	TACGCGTCCCGGGGCGGTACCATGGCGGCCAAAATCGG
Ma1-JW772-R	ACGAAAGCTCTGCAGGTCGACTTAGTTCTTCAACCGCAA
